# Generation of a High‐Precision Whole Liver Panorama and Cross‐Scale 3D Pathological Analysis for Hepatic Fibrosis

**DOI:** 10.1002/advs.202502744

**Published:** 2025-03-24

**Authors:** Xiaochuan Zhang, Weicheng Yang, Xiaoliang Li, Yanli Zhao, Zongneng Xie, Shuangqu Li, Yue Zeng, Xiaoxu Hao, Xiaohong Xin, Yu Zhang, Zixuan Feng, Hualiang Jiang, Zhaobing Gao, Xianzhen Yin

**Affiliations:** ^1^ Center for MOST and Image Fusion Analysis Shanghai Institute of Materia Medica Chinese Academy of Sciences Shanghai 201210 China; ^2^ CAS Key Laboratory of Drug Research Shanghai Institute of Materia Medica Chinese Academy of Sciences Shanghai 201203 China; ^3^ School of Chinese Materia Medica Nanjing University of Chinese Medicine Nanjing 210023 China; ^4^ Lingang Laboratory Shanghai 201602 China; ^5^ Zhongshan Institute of Drug Discovery Institution for Drug Discovery Innovation Chinese Academy of Science Zhongshan 528400 China

**Keywords:** 3D imaging, hepatic fibrosis, micro‐optical sectioning tomography, pathological atlas, whole liver

## Abstract

The liver harbors complex cross‐scale structures, and the fibrosis‐related alterations to these structures have a severe impact on the diverse function of the liver. However, the hepatic anatomic structures and their pathological alterations in the whole‐liver scale remain to be elucidated. Combining the micro‐optical sectioning tomography (MOST) system and liver Nissl staining, a first high‐precision whole mouse liver atlas is generated, enabling visualization and analysis of the entire mouse liver. Thus, a detailed 3D panorama of CCl4‐induced liver fibrosis pathology is constructed, capturing the 3D details of the central veins, portal veins, arteries, bile ducts, hepatic sinusoids, and liver cells. Pathological changes, including damaged sinusoids, steatotic hepatocytes, and collagen deposition, are region‐specific and concentrated in the pericentral areas. The quantitative analysis shows a significantly reduced diameter and increased length density of the central vein. Additionally, a deep learning tool is used to segment steatotic hepatocytes, finding that the volume proportion of steatotic regions is similar across liver lobes. Steatosis severity increases with proximity to the central vein, independent of central vein diameter. The approach allows the cross‐scale visualization of multiple structural components in liver research and promotes pathological studies from a 2D to a 3D perspective.

## Graphical abstract

Using the MOSTtechnology and whole‐liver Nissl staining method, a multi‐structural 3D pathological panorama of liver fibrosis is obtained. The damaged sinusoids, steatotic hepatocytes, and collagen deposition are all concentrated in pericentral areas. AI segmentation and analysis show that hepatocyte steatosis severity increased with distance from central veins, independent of vein diameter.

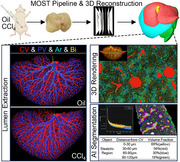

## 1 Introduction

The mammalian liver harbors multiple essential functions including manufacturing required chemicals and detoxifying redundant substances in the body.^[^ ^1^ ^]^ It is responsible for generating essential biomolecules like ammonium, fatty acids, amino acids, and glucose, as well as metabolizing harmful compounds.^[^ ^2^ ^]^

The liver possesses intricate and hierarchical segmental organization. In mice, the liver is separated into the caudate, left (biggest), right (bisected), and medial lobes.^[^ ^3^ ^]^ Liver lobules, the basic functional units of the liver, are roughly hexagon‐shaped structures with a central vein in the center and a portal vein at each intersection.^[^ ^4^ ^]^ The hepatic artery and the bile extend accompanied by the portal veins. The liver is supplied by the hepatic portal vein (75%) and hepatic artery (25%), allowing nutrient‐rich blood from the intestine to enter the liver lobules.^[^ ^2^ ^]^ The blood then flows through small channels called sinusoids that are lined with primary liver cells. After being depleted of nutrients and oxygen by the hepatocytes, the blood leaves the lobule through the bile duct and central vein.^[^ ^5^ ^]^ The direction of the bile flow is opposite to that of the lobular blood flow.^[^ ^6^ ^]^ Hepatocyte‐produced bile is collected by bile canaliculi, which branch out and combine into the intrahepatic bile ducts. The gallbladder is used for short‐term bile storage, or it can be emptied straight into the duodenum.^[^ ^7^ ^]^

Given the vital functions in production, recycling, storage, and waste management, it is not surprising that impaired liver function is associated with an extensive variety of illnesses. The liver diseases including infections such as hepatitis, cirrhosis (scarring), cancers, and damage by medications or toxins are major causes of illness and death worldwide.^[^ ^8^ ^]^ Various murine models are currently utilized for studying acute and chronic pathological processes of the liver, as well as assessing the effectiveness of new therapeutic approaches. Carbon tetrachloride (CCl_4_) is commonly used for free radical‐induced liver injury as many experimental and clinical studies consider it as a classical hepatotoxic agent that induces liver cirrhosis, fibrosis, and necrosis.^[^ ^9^ ^]^ The growing accessibility of high‐resolution small animal imaging techniques provides researchers with the ability to accurately identify and characterize liver pathologies.

Classical 2D slice histology, the gold standard for investigations of liver anatomy, is still flourishing together with 3D visualization methods due to their wide application and high‐resolution properties. The 2D histological slices provide a wealth of information on the morphological and functional characteristics of biological tissues, which play an important role in understanding diseases and assisting diagnosis.^[^ ^10^ ^]^ But 3D volume imaging is more powerful for conducting spatial analysis, exploring spatial relationships between microscopic anatomical objects, and discovering global spatial patterns. Non‐invasive imaging techniques such as computed tomography (CT),^[^ ^11^ ^]^ magnetic resonance imaging (MRI),^[^ ^12^ ^]^ and positron‐emission tomography (PET)^[^ ^13^ ^]^ are routine for clinical imaging to in‐depth investigate complex hepatic anatomy, but show inadequate resolution at cellular and subcellular level. It is worth mentioning that, using synchrotron radiation micro‐computed tomography, the anatomy of the hepatic sinusoids in both normal and CCl_4_‐damaged mice livers can be determined three‐dimensionally.^[^ ^14^ ^]^

Coupled with the specific labeling, the large‐volume imaging techniques were applicable to image and analyze hepatic cells or tubular structures. The light‐sheet microscopy combing with the iDISCO method, which enables the whole‐tissue immunolabeling and optical clearing of intact tissues, was employed for visualizing microvasculature and tumor cells in murine colorectal liver metastases,^[^ ^15^ ^]^ as well as sympathetic innervations in murine liver disease.^[^ ^16^ ^]^ The fluorescent micro‐optical sectioning tomography (fMOST) system was also adopted to imaging intact liver lobe of Tek‐Cre:Ai47 mice to acquire the vessel structures and cytoarchitecture information with single‐cell resolution based on propidium iodide staining and vessel‐specific labeling.^[^ ^17^ ^]^ Owing to the lack of specific markers for hepatic lymphatic endothelial cells (LyECs), a method for the spatiotemporal sequential injection of antibodies (STSI‐Ab) was designed to selectively label hepatic LyECs in vivo, and then the 150‐µm slices of labeled liver lobes were imaged using HD‐fMOST.^[^ ^18^ ^]^ These studies substantially enriched our knowledge of the anatomy and pathological characteristics of the liver. A comprehensive investigation of structural and morphological characterization of hepatic components as much as possible in the whole liver will greatly facilitate an understanding of the precise architecture of the normal liver and pathogenesis of liver disease.

Employing the reflected bright‐field imaging technique of MOST with a whole‐organ Nissl staining method, our previous study demonstrated the capability to distinguish different structures that include cells, blood vessels, and airways based on the differences in gray values and morphologies.^[^ ^19^ ^]^ Here, we are able to visualize the central vein, portal vein, artery, bile duct, sinusoid, and hepatocyte simultaneously in mouse liver based on MOST system and whole‐liver Nissl staining. This research provides a novel approach to clearly present various structural information in a whole mouse liver simultaneously, which will elevate the pathological research from a 2D to a 3D perspective.

## 2 Results

### 2.1 Acquisition of a Whole Mouse Liver Atlas

**Figure 1:**
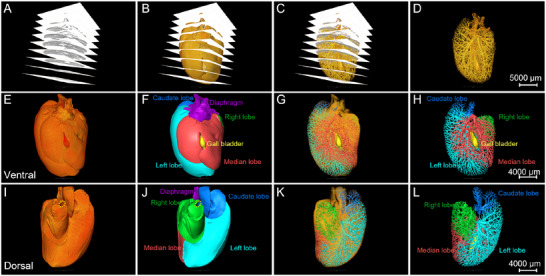
High‐resolution whole liver atlas provides global view of liver lobes and vessels in C57BL/6 mouse. A–D) High‐quality coronal image sequence of the intact liver from a 2‐month‐old male C57BL/6 mouse. Based on these high‐quality images, the surface (B) and inner vessels (C,D) of whole mouse liver can be visualized. Scale bar, 5000 µm. E–H) Ventral view of 3D‐reconstructed whole mouse liver, showing the surface (E,F) and inner vessels (G,H) of different segmented lobes including the median lobe, left lobe, right lobe, and caudate lobe. The gall bladder is located between the left and right medial lobe. The structure in purple color was the thoracic diaphragm. Scale bar, 4000 µm. I–L) Dorsal view of 3D‐reconstructed whole mouse liver, showing the surface (I,J) and inner vessels (K,L) of the four segmented main liver lobes including the median lobe, left lobe, right lobe, and caudate lobe. The dotted line in the (I,J) denotes the liver hilus. Scale bar, 4000 µm.

Using the MOST system, whole mouse liver datasets were acquired at a resolution level of 0.35 µm × 0.35 µm × 2 µm from a 2‐month‐old male C57BL/6 mouse. Representative images of the different coronal sections of the whole‐liver Nissl staining datasets were shown in Figure S1 (Supporting Information). From these 2D images, the mouse liver harbor four major lobes consisting of the median lobe, the left lobe, the right lobe and the small leaf shaped caudate (Figure S1A–I, Supporting Information). The gall bladder is sandwiched between the left median and right median lobe (Figure S1C,D, Supporting Information). And the vessels were obviously displayed in the liver parenchyma. Based on ≈9000 high‐quality slices, the whole mouse liver was reconstructed (**Figure** **1**A–D). From the ventral view (Figure 1E–H) and dorsal view (Figure 1I–L), both the high‐quality rendering and surface reconstruction of an intact mouse liver illustrated the four liver lobes and the gall bladder and their inner vessels. The surfaces and inner vessels of these liver lobes were segmented and depicted in different colors: the median lobe in red, left lobe in cyan, right lobe in green, and caudate lobe in blue. These results demonstrate the ability of clear visualization and analysis of the whole mouse liver.

### 2.2 Generation of Pathological Datasets in Liver Fibrosis

**Figure 2:**
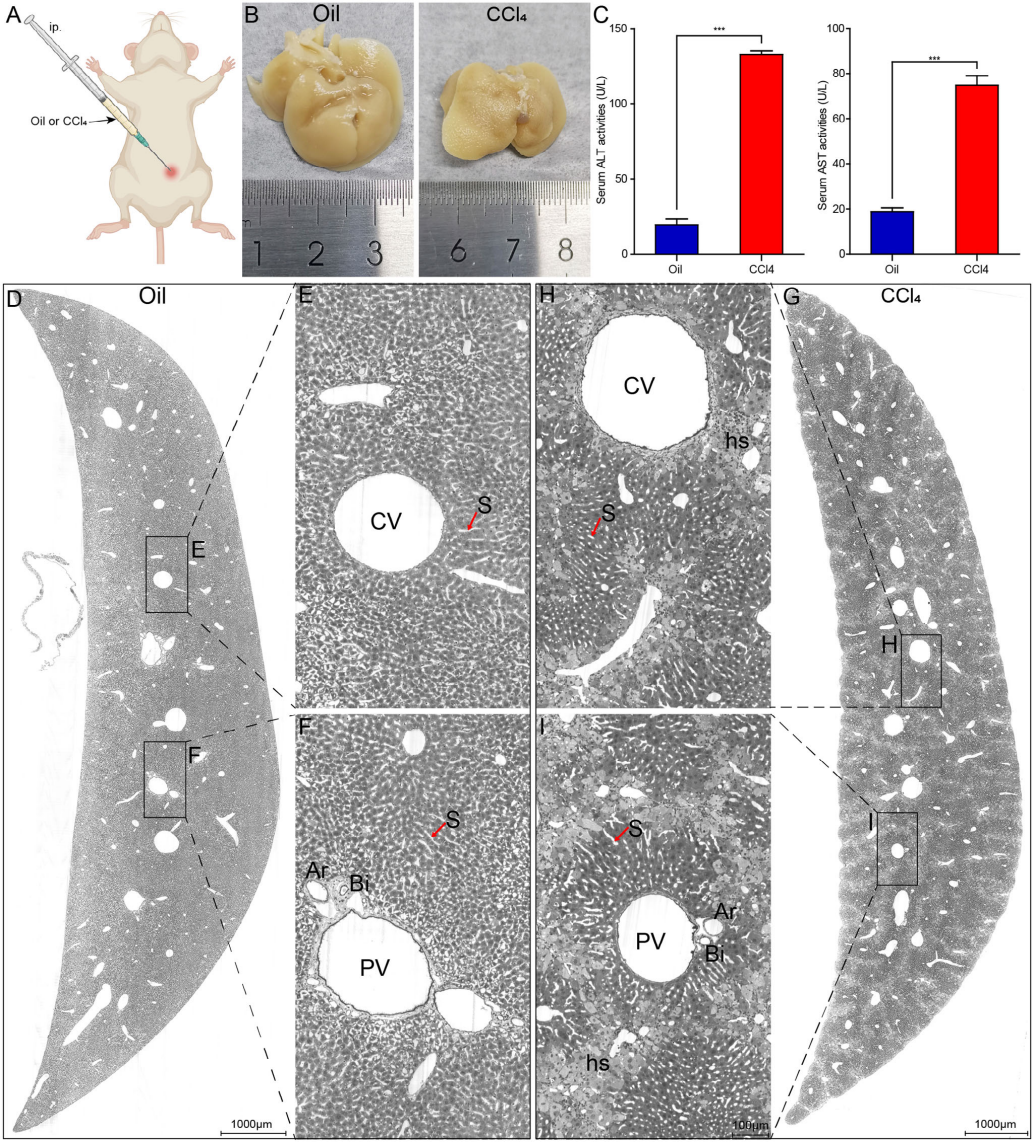
High‐resolution pathological atlas shows the typical injury and multiple signals of liver structures. A) The CCl_4_‐treated and oil‐treated mice were injected intraperitoneally with 10% CCl_4_ diluted in corn oil or corn oil alone twice a week for 6 weeks. B) Macroscopic appearance of the livers from both the oil and CCl_4_ group. C) Serum biochemical parameters (ALT and AST) analysis. Elevation of ALT and AST as a fibrotic sign in CCL_4_ group was observed. D–G) Representative coronal images of Nissl‐staining left liver lobes from both the oil and CCl_4_ groups. The jagged liver edge, and hepatocyte steatosis (hs) in the CCL_4_ group were quite obvious. The higher gray‐scale signals of central veins (CV), portal veins (PV), arteries (Ar), bile ducts (Bi), and hepatic sinusoids (S, red arrow), as well as the lower gray‐scale signals of liver cells can be clearly identified. The bile ducts and arteries were paralleled closely with the portal veins, and the epithelial cells of bile ducts were much darker and thicker than that of the accompanying arteries. Scale bar in (D,G), 1000 µm. Scale bar in (E,F,H,I), 100 µm.

To verify the performance of our method to visualize the liver damage, we conducted the Nissl staining and subsequent MOST imaging on CCl_4_‐induced liver fibrosis mice and control mice (corn oil treated). The mice were intraperitoneally injected with corn oil or 10% CCl_4_ diluted in corn oil for 6 weeks (**Figure** **2**A). Then, the livers were harvested after cardiac perfusion, and the levels of serum alanine aminotransferase (ALT) and aspartate aminotransferase (AST) were measured. From the macroscopic appearance of the mouse livers, we found that compared with oil group, the liver in CCl_4_‐treated group exhibited a coarser surface characterized by a granular and uneven pattern, namely surface nodularity (Figure 2B). This phenomenon revealed that the liver surface in hepatic fibrosis may exhibit an altered texture with an excessive deposition of scar tissue. The serum ALT increased by ≈7‐fold, and serum AST rose ≈4‐fold after CCl_4_ administration, indicating the liver function was impaired by the CCl_4_ administration (Figure 2C). Representative Nissl‐staining images of the coronal sections in the oil and CCl_4_ group were shown in Figure 2D–I. The liver of oil group mice had normal lobular structure with central vein and radial hepatic cord (Figure 2E,F), however, the liver following the CCl_4_ administration exhibited with severe hepatocyte steatosis surrounding the central vein (Figure 2H,I). Among the regions of hepatocyte steatosis, there are abundant smaller cells, which are probably inflammatory cells. And the edge of the liver lobe in the CCl_4_ group exhibited a jagged or serrated appearance, in contrast to the smooth edge of the liver lobe in the oil group.

Besides, H&E and Masson's trichrome staining were also used to validate the histopathological alterations and collagen deposits in the liver slices. As shown in Figure S2A,C (Supporting Information), the H&E staining of the control group showed a normal histological appearance, whereas those of CCl_4_ group exhibited obvious hepatic steatosis as same as demonstrated in Nissl staining images. Only a small amount of collagen was present in the oil group in the portal and central areas (Figure S2B, Supporting Information). In contrast to the oil group, abundant collagen fibers in the CCl_4_ group (in blue color) surrounded the central vein and were consistent with the distribution of damaged liver cells (Figure S2D, Supporting Information). According to the Masson's trichrome staining, the regions of collagen deposition overlapped with the regions of hepatocyte steatosis. These results indicate that Nissl staining can also provide information comparable to H&E staining.

Observing both the oil and CCl_4_ group, we found that the high gray‐scale signals of central veins, portal veins, arteries, bile ducts and hepatic sinusoids, as well as the lower gray‐scale signals liver cells could be legibly discernible (Figure 2D–I). It is worth mentioning that the portal veins were accompanied by the bile duct, artery and other lumen structures, but there were no other lumen structures around the central veins (Figure 2E,F,H,I). This characteristic phenomenon makes it easy to distinguish between the central and portal veins. Additionally, the bile ducts were formed by much darker and thicker epithelial cells, and roughly equivalent in size to its accompanying arteries (Figure 2F,I; Figure S3A,B, Supporting Information). From the rendering results of the portal triad (Figure S3A,B, Supporting Information), in addition to the artery and bile duct, the signals of peribiliary vascular plexus and lymphatic vessel can also be identified. Instead of extracting the peribiliary vascular plexus and lymphatic vessels over the entire liver lobe, we reconstructed the normal portal triad in the local area due to the huge workload for extraction and the limitation of the resolution. The 3D reconstruction of the portal triad showed that the peribiliary vascular plexus was around the bile duct, and connected the artery and the portal vein (Figure S3C–F, Supporting Information). And the morphology of lymphatic vessel was quite irregular (Figure S3G,H, Supporting Information), which was distinct from the tube‐like structures in a previous study.^[^ ^17^ ^]^ These discernible signals of multiple structures enable us to simultaneously visualize several pathological features at submicron resolution of 3D whole‐liver scale, especially in the same sample.

### 2.3 Visualization and Characterization of Complex Tubular Structures in Intact Left Liver Lobe

To allow for a comprehensive evaluation of hepatic damage, we conducted 3D reconstruction of the entire liver from the CCl_4_ model mice. The whole‐liver surfaces of CCl_4_ model mice (Figure S4A–D, Supporting Information) exhibited prominent nodularity in all lobes. The volume rendering of the 30‐µm‐thick coronal slices across different lobes in the CCl_4_ mouse was also performed (Figure S4E,F, Supporting Information). From the enlarged view of these coronal slices, we observed that the all the liver lobes were filled with steatotic hepatocytes around the central veins (Figure S4E_1‐5_, F_1‐5_, Supporting Information). These results revealed that there were no evident differences in the main pathological characteristics across the liver lobes of CCl_4_ mouse.

**Figure 3:**
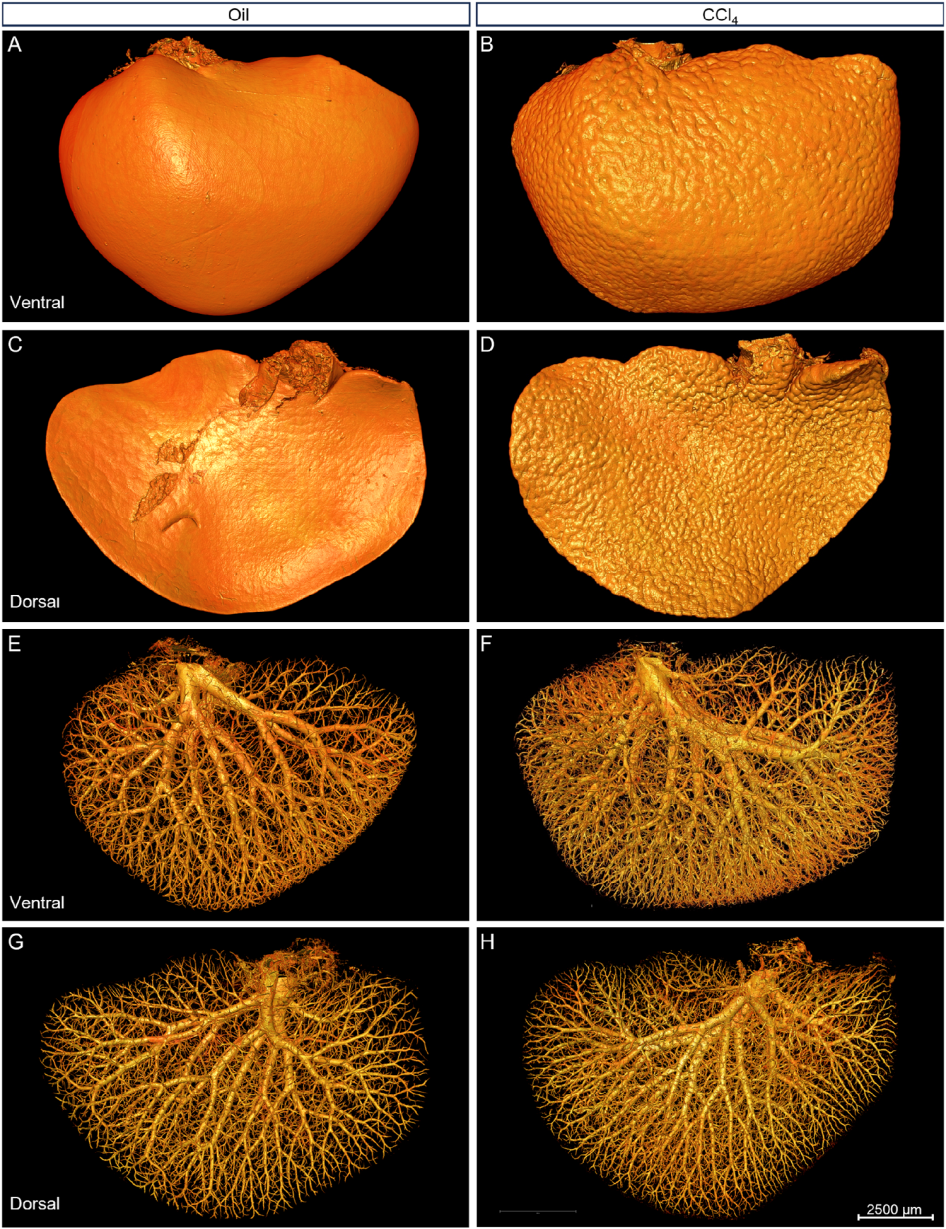
Comparison of the surface and vasculature network between the oil and CCl_4_ group. A–D) Ventral (A,B) and dorsal (C,D) view of the 3D reconstructed liver surfaces of the left lobes from both the oil (A,C) and CCl_4_ (B,D) groups. The surface of CCl_4_‐treated mice was full of nodules, compared with the smooth surface in the oil‐treated mice. E–H) Ventral (E,F) and dorsal (G,H) view of the vasculature network of the left lobes from both the (E,G) and CCl_4_ (F,H) group. The vasculature in the CCl_4_ group was observed with much denser and smaller vessels in the terminal end of branches, compared with the oil group. Scale bar, 2500 µm.

Given the similar pathological characteristics among the liver lobes, and to reduce the computational burden, we performed whole‐mount Nissl staining and obtained MOST‐generated datasets on the left liver lobes (0.35 µm × 0.35 µm × 1 µm), which were a frequent site of sampling for histology. To ascertain the precise pathological alternations in liver fibrosis, we made high‐precision reconstruction of the intact left liver lobe according to the MOST‐generated Nissl staining datasets. The high‐precision rendering of liver surface (**Figure** **3**A–D) and inner vessels (Figure 3E–H) illustrated the differences between the oil and CCl_4_ group more clearly in intact liver lobe scale. From both the ventral view (Figure 3A,B) and dorsal view (Figure 3C,D), the surface nodularity was quite prominent in the CCl_4_ group, and there was no nodularity in the oil group. These findings coincided with previous results as shown in Figure 2B,D. As shown in Figure 3E–H, the vessels in the CCl_4_ group appeared much denser than those in the oil group.

**Figure 4:**
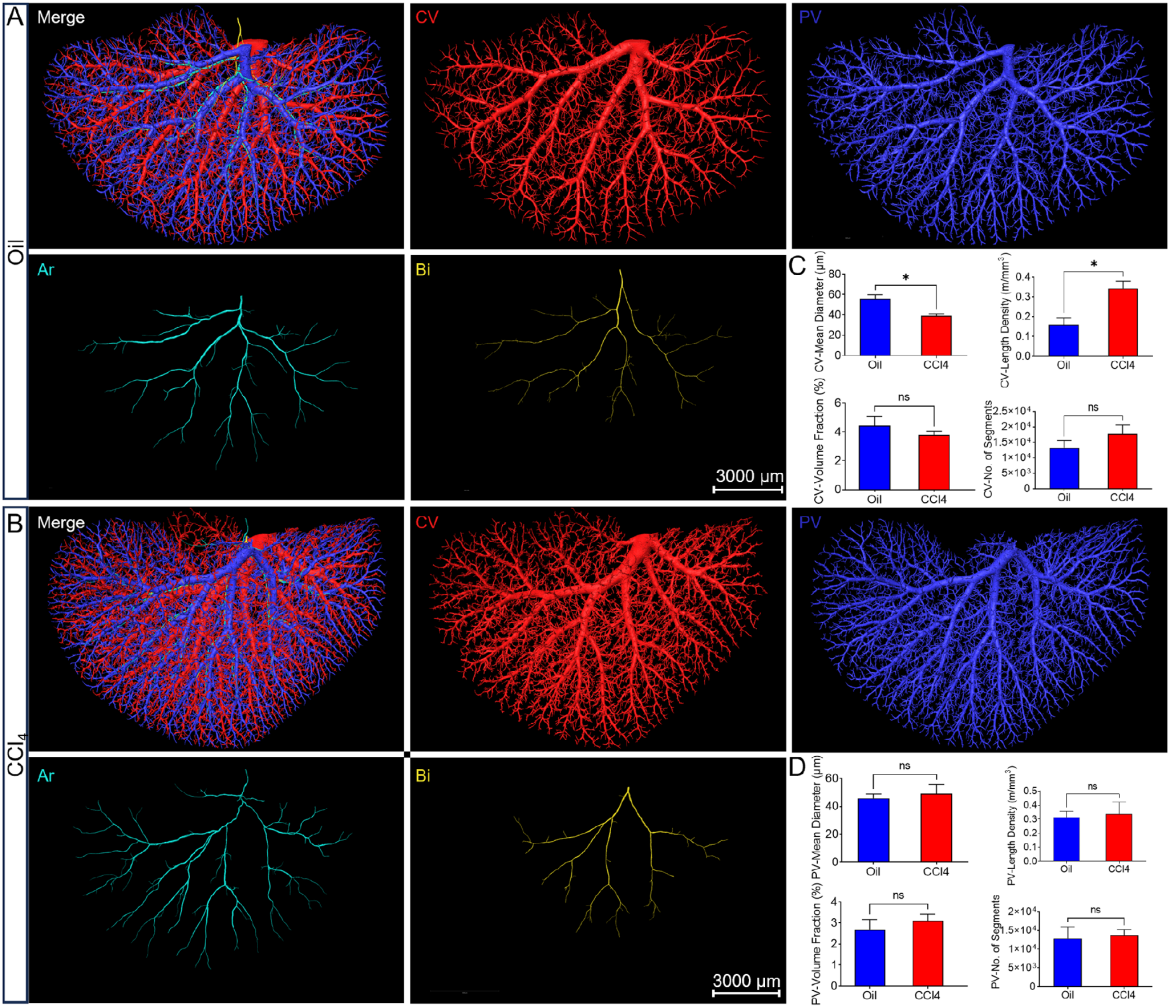
Segmentation and quantitative analysis of the tubular structures of in intact left liver lobe. A,B) Doral view of the tubular structures including the central vein (CV), portal vein (PV), artery (Ar), and bile duct (Bi) in the oil and CCl_4_ group. Scale bar, 3000 µm C) Quantitative analysis of the central veins from both the oil and CCl_4_ group, exhibiting the changes in the mean diameter, length density, number of segments, and volume fraction. D) Quantitative analysis of the portal veins from both the oil and CCl_4_ groups, exhibiting the changes in the mean diameter, length density, number of segments, and volume fraction. (Unpaired *t*‐test; ^*^ *p* < 0.05; ns = non‐significant, *p* > 0.05; n = 3 mice per group).

To further define the changes in tubular structures, we segmented the central veins and portal veins according to the connectivity of these tubular structures and region growing algorithm in Amira 3D software. Given the relatively small signals of the arteries and bile ducts, which are also in close proximity to other structures at the current resolution, it becomes challenging to extract the arteries and bile ducts based on regional connectivity alone. The arteries and bile ducts were manually identified and segmented on the basis of the different morphology of their epithelial cells. As observed from the dorsal view in **Figure** **4** A,B, the central veins, portal veins, arteries, and bile ducts of both the oil and CCl_4_ group were extracted and reconstructed. The arteries and bile ducts were found spatially paralleling with the portal veins (Figure S5, Supporting Information), which could also be verified from Figure 2F,I. The quantitative analysis showed that there were no significant changes concerning the morphological parameters of the portal veins (Figure 4C,D). But the length density of the central veins had significantly increased and the mean diameter of the central veins had significantly decreased (Figure 4C). Besides, the quantitative results suggested a tendency for an increase in the number of central vein segments and a decrease in the central vein volume fraction in the CCl4 group, yet the difference was not statistically significant (Figure 4D). These results implied the increased small vessels due to hepatic sinusoidal capillarization.

### 2.4 Region‐Specific Damage of Hepatic Sinusoid and Cell in Liver Fibrosis

**Figure 5:**
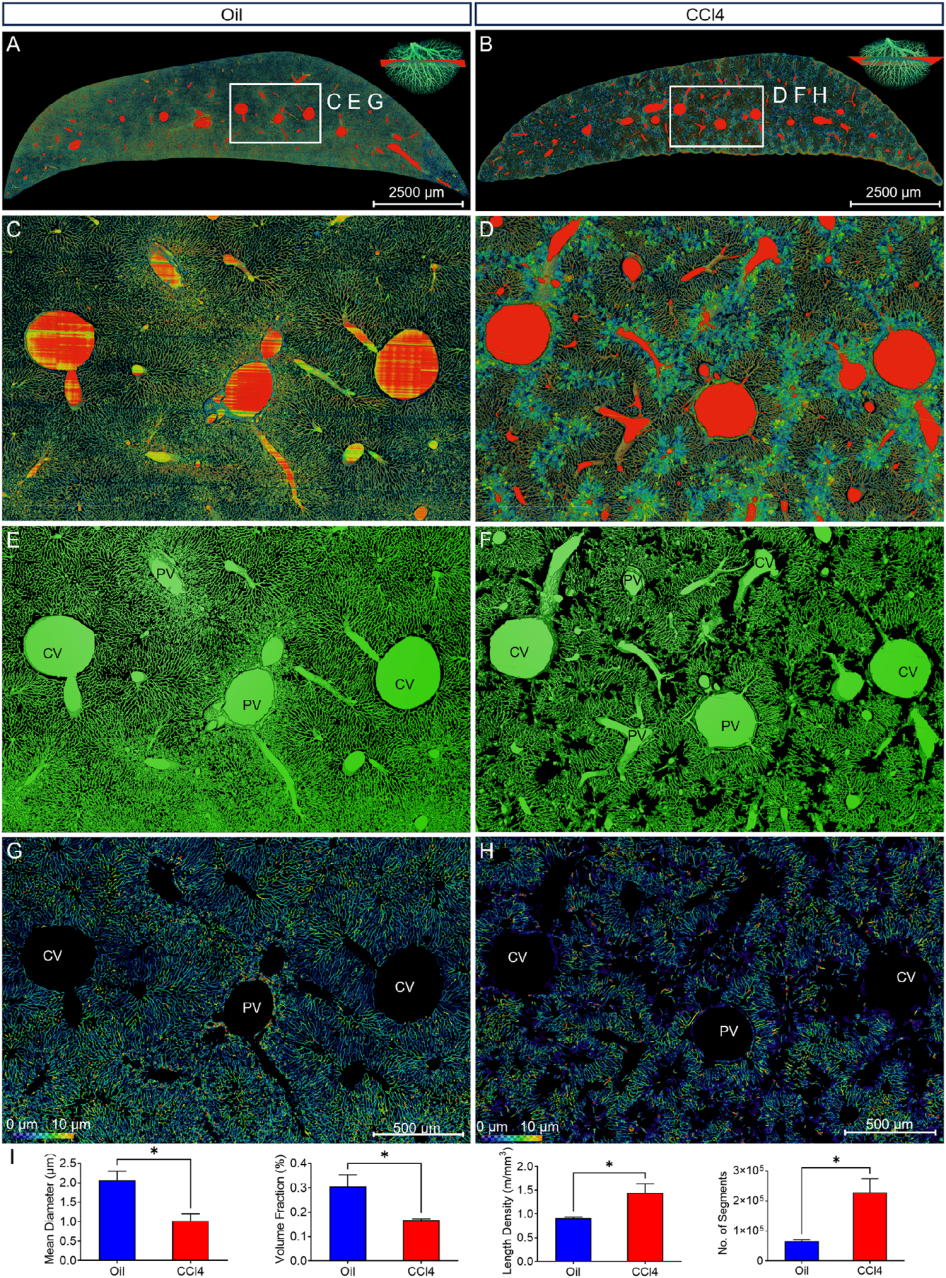
Simultaneous visualization of vessels, hepatic sinusoids, and steatotic hepatocytes in representative coronal slices. A,B) Volume rendering of the 30‐µm‐thick coronal slices across similar anatomical position in both the oil and CCl_4_ group. Enlarged views of the boxes in A, B were shown in C, E, G and D, F, H, respectively. Scale bar, 2500 µm C,D) Simultaneous visualization of vessels, hepatic sinusoids, and steatotic hepatocytes in both the oil and CCl_4_ group, showing no steatosis in the oil group and abundant steatotic hepatocytes in the pericentral areas of CCl_4_ group. E,F) Simultaneous visualization of vessels and hepatic sinusoids in both the oil and CCl_4_ group, showing the severe damage of pericentral sinusoids with noticeable vacancies in the network of CCl_4_ mice. G,H) Segmentation and skeletonization of the hepatic sinusoids with sinusoid diameter coded in color, demonstrating more smaller sinusoids in diameter after the CCl_4_ administration. I) Quantitative analysis on the hepatic sinusoids of the oil and CCl_4_ group using morphological parameters including mean diameter, volume fraction, length density, and number of segments (Unpaired *t* test; ^*^ *p* < 0.05; ns = non‐significant, *p* > 0.05; n = 3 mice per group). Scale bar in (C,E,G), 500 µm. Scale bar in (D,F,H), 500 µm.

To provide local insights into the liver structures, we applied a volume rendering of the 30‐µm‐thick coronal slices across similar anatomical positions in both the oil and CCl_4_ groups. The coronal views simultaneously illustrated the information of vessels, sinusoids, and steatotic hepatocytes (**Figure** **5**A,B). The enlarged views of Figure 5A,B shows that compared with the normal structure of hepatic sinusoids, the hepatic sinusoids in the CCl_4_ group were obviously damaged and occupied by steatotic hepatocytes (Figure 5C,D). The damaged regions were mainly the pericentral sinusoids rather than the periportal sinusoids in the CCl_4_ group. And the pericentral sinusoids in the CCl_4_ showed a sparser and more disconnected network compared with that in the oil group (Figure 5E,F). The periportal sinusoids appeared with the normal connected network in both the oil and CCl_4_ groups (Figure 5E,F). From the rendering images of cells (Figure S6A–F, Supporting Information), the liver cells in the oil group were dense and regularly arranged in both the pericentral and periportal areas (Figure S6E–G, Supporting Information), while the liver cells in the CCl_4_ group were sparse and appeared with abundant smaller cells around the central veins (Figure S6F1, Supporting Information). These cells with smaller cells are likely to be inflammatory cells. It is easy to distinguish the bile ducts from the arteries in terms of endothelial cell morphology, with bile duct endothelium being thicker and denser than that of arteries (Figure S6G,H, Supporting Information). By viewing only sinusoid images (extraction based on gray values and threshold segmentation) with diameters coded by pseudo‐colors, the clear sinusoid abnormality was found in the CCl_4_ group, whereas the sinusoids in the oil group were quite normal and dense (Figure 5G,H). We also performed a quantitative analysis on the extracted 3D label data of the oil and CCl_4_ liver using morphological parameter. According to Figure 5I, the mean diameter and volume fraction of the CCl_4_ group were significantly declined, compared with that of oil group. These results exhibited a sharp reduction in the sinusoidal volume, which was caused by the disruption of the sinusoid network due to CCl₄ treatment. Besides, the length density and number of segments of the CCl_4_ group were remarkably increased, which might be induced by the hepatic sinusoidal capillarization.

**Figure 6:**
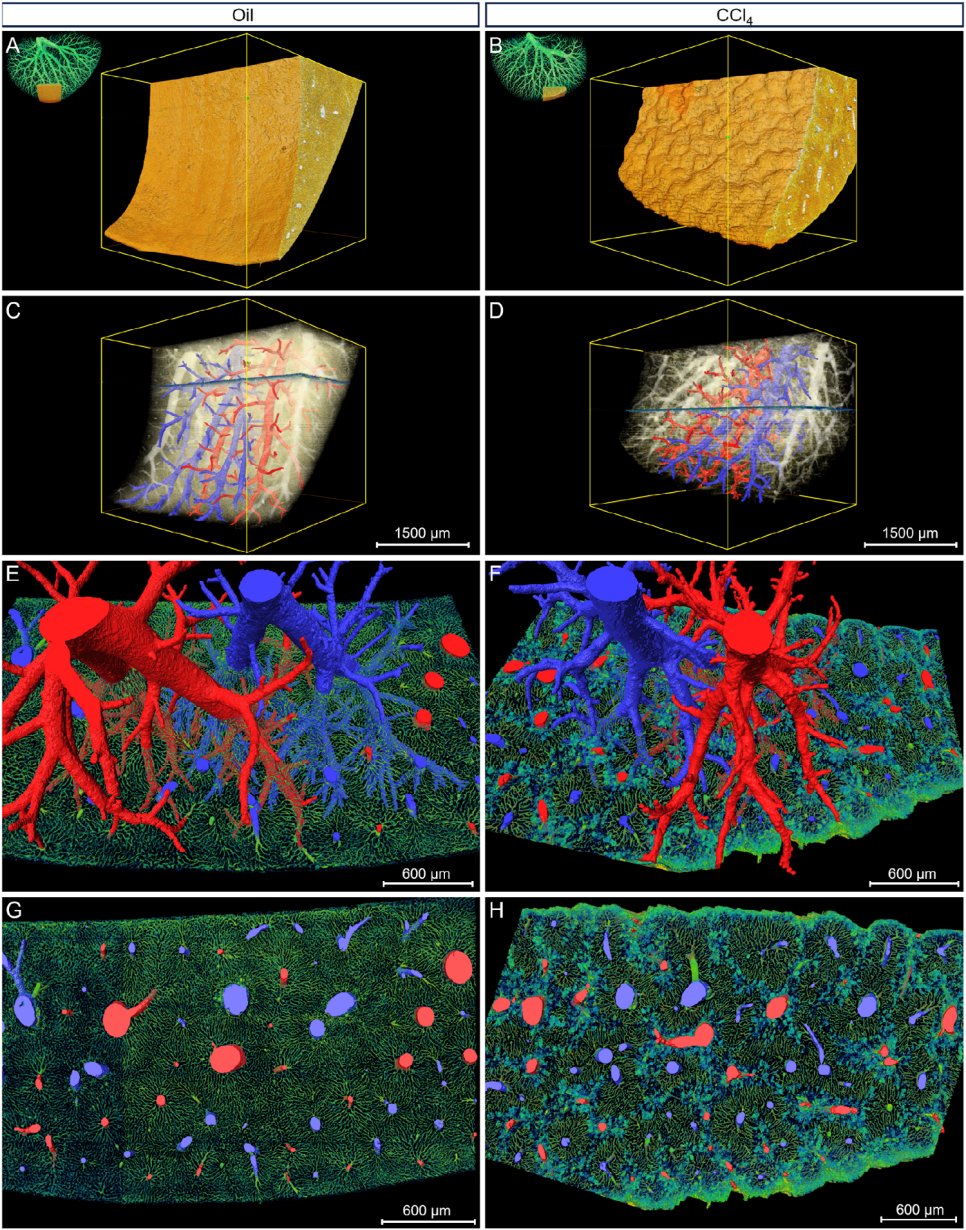
Spatial position of the damaged hepatic sinusoids, and steatotic hepatocytes. A,B) Volume rendering of the data blocks showing the size and position of the ROIs. C,D) 3D reconstruction demonstrating the veins of the ROIs, and the position of 30‐µm‐thick coronal slices and veins in E‐H. E–H) Simultaneously displaying the rendering of 30‐µm‐thick coronal slices, and the segmented central vein (in red) and portal vein (in blue) to show the region‐specific distribution of damaged sinusoids and steatotic hepatocytes. Scale bar in (A,C), 2500 µm. Scale bar in (B,D), 2500 µm. Scale bar in (E–H), 600 µm.

We also selected regions of interest (ROI) from the liver lobes with the size and position as shown in **Figure** **6**A–D denoted the position of coronal slices and veins in the Figure 6E–H. In Figure 6E–H, we segmented the individual branch of central vein (in red color) and portal vein (in blue color), and reconstructed the liver structures of the coronal plane. Obviously, the hepatic sinusoids in the oil group were regular and normal either around the portal vein or the central vein (Figure 6E,G). By contrast, the damaged hepatic sinusoids and steatotic hepatocytes in the CCl_4_ group were surrounding the central veins. These results further supported the region‐specific damages of pericentral sinusoids and their neighboring hepatic cells from a 3D perspective.

### 2.5 Automatic Segmentation and 3D Visualization of Steatotic Hepatocyte in Liver Fibrosis

**Figure 7:**
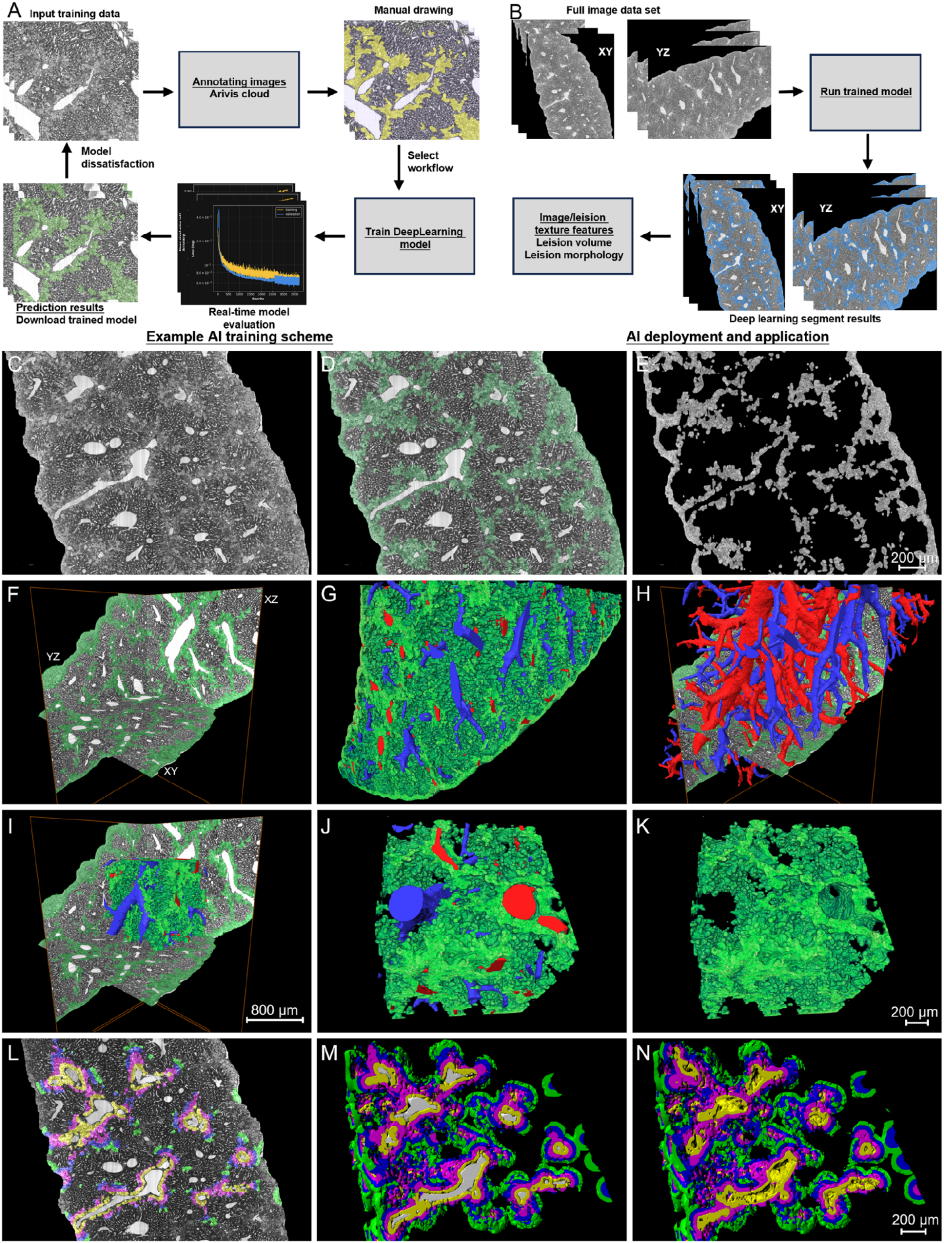
AI segmentation and visualization analysis of steatotic hepatocyte in the CCl4 mouse. A) Upload training data to Arivis cloud and manually draw the target and background objects to train specific deep learning models for the segmentation of steatotic hepatocyte. B) The trained model was deployed for predicting cell segmentation in the whole data block, allowing for high‐throughput mining of specific tissue pathology. C–F) Representative orthogonal slices showing the segmented steatotic hepatocyte. G,H) Volume rendering of the segmented steatotic hepatocytes and the surface reconstruction of the corresponding central/portal veins. The central vein was in red color; and the portal vein was in blue color. I–K) Visual reconstruction of a smaller region in the center of the data block. L–N) Quantitative analysis of the steatotic regions according to the distances from the surface of central veins. Scale bar in (C–E), 200 µm. Scale bar in (F–I), 800 µm. Scale bar in (J,K), 200 µm. Scale bar in (L–N), 200 µm.

Since the areas of collagen deposition overlapped with those of steatotic hepatocytes, studying the steatotic areas can also provide insights into the distribution and other aspects of the collagen deposition areas. To obtain a detailed description of the abnormal hepatocytes, we selected the data block as shown in Figure 6B to investigate the distribution of steatotic hepatocytes and their interaction with other structures. The steatotic hepatocytes were identified and segmented automatically using deep learning models in ZEISS Arivis software. First, a training data block was cropped and uploaded to the Arivis cloud, and then a trained individual conducted manual labeling and segmentation of injured hepatocytes on the uploaded data to achieve a trained deep learning model. Figure S7 (Supporting Information) shows that the accuracy and reliability of the trained model was sufficient for next automatic segmentation after ≈3500 training iterations. And the next step, we exerted the automatic segmentation based on downloaded deep learning model on the local Arivis Vision 4D software (**Figure** **7**A,B, see the Experimental Section). Figure 7C–F shows the representative images of labeled data, and the labeled data were displayed with transparent green color and colocalized with the regions of steatotic hepatocytes. The extracted steatotic hepatocytes were obtained via multiplying the labeled image by the grayscale image (Figure 7E). Based on these segmented images, we reconstructed the steatotic hepatocytes of the whole data block three‐dimensionally (Figure 7G). These injured hepatocytes (in green color) were distributed alongside the central veins (Figure 7G,H, in red color) and led to severe disrupt of pericentral sinusoids (Figure 7D,F). By quantitative calculation, the total volume of the data block and labeled hepatocyte regions was 8.53 and 2.62 mm^3^ respectively, and the volume fraction of injured hepatocyte regions was 30.76% (**Table** **1**). The high proportion of injured hepatocyte volume indicated that the liver was disrupted severely and the collagen deposition was quite widespread.

**Table 1:** Morphological parameters of the segmented region of steatotic hepatocytes and the data block in Figure 7G.

Labeled steatotic hepatocyte	Length3d [µm]	Thickness3d [µm]	EqDiameter [µm]	Volume3d [µm^3^]	Shape_VA3d	Perimeter [µm]
Mean	24.6367321	13.82653046	16.25183105	579610.125	3.967603922	39305.74219
Min	1.094837904	0	1.24070096	1	0.23973316	1
Max	4243.73877	1780.286621	1706.519897	2 602 150 400	11604.29883	173 112 816
Median	19.54961014	11.6167984	14.16133308	1487	1.209451199	544.5
Variance	4221.467285	752.7531738	721.7283325	1.49509E+15	29726.45703	6.61689E+12
Kurtosis	3923.92041	3791.880127	3457.719971	4523	4523.382813	4523.001465
Skewness	60.51002121	58.97241974	55.05527115	67.26811981	67.27239227	67.26813507
Total	1.12E+05			2.62E+09		1.78E+08
Volume fraction				30.76%		
Data block	Length3d (µm)	Thickness3d (µm)	EqDiameter (µm)	Volume3d (µm^3^)	Shape_VA3d	Perimeter (µm)
1	4287.6	1779.58	2535.26	8.53E+09	2.79639	20 327 413

Note: Data are based on measurements from the ROI shown in Figure 7G.

To examine pathological alterations in all liver lobes and explore potential inter‐lobe differences, we also segmented the whole liver using abovementioned deep learning model. The representative images of coronal sections demonstrating the identification and segmentation results of the steatotic regions were shown in Figure S8A–D (Supporting Information). The quantitative results exhibited that the percentages of volume of the steatotic regions were almost identical in the left lobe (32.58%), right lobe (32.02%) and caudate lobe (31.85%), except for the median lobe (26.95%) (**Table** **2**). Upon investigation, we found that some steatotic regions in the median lobe were not correctly identified and segmented (Figure S8E,F, Supporting Information; Table 2). This was due to insufficient perfusion, which led to inadequate filling of the hepatic sinusoids, thereby resulting in atypical pathological features surrounding the steatotic regions. Taking this factor into account, the volume ratios of the steatotic regions in each liver lobe should have minimal differences originally.

**Table 2:** The comparison of the volume of labeled steatotic regions in different liver lobes.

Liver Lobes	Steatotic Region Volume [10^10^ µm3]	Lobe Volume [10^10^ µm3]	Percentage [%]
Left Lobe	7.0941	21.7757	32.58
Median Lobe	6.1507	22.8187	26.95
Right Lobe	6.2186	19.4200	32.02
Caudate Lobe	1.5057	4.7279	31.85

Note: Data are based on measurements of whole liver of CCl4 mouse. The steatotic regions of different lobes in the whole liver were segmented using the Deep Learing model.

To more clearly demonstrate the association between steatotic hepatocytes and vessels, we performed a visual reconstruction of a smaller region in the center of the data block. Figure 7I–K shows the position of the ROI (943 × 843 × 1129 µm^3^). From the high‐precise reconstruction of the smaller ROI, the central veins were closely surrounded by the steatotic hepatocytes, in contrast to no steatotic hepatocytes around the portal veins. To demonstrate more clearly the degree of hepatocyte degeneration at different distances from the central veins, we re‐segmented the steatotic regions at different distances from the surface of central veins with different colors. As shown in Figure 7L, the distribution pattern of steatotic hepatocytes was analyzed on three distance ranges: 0–30 µm (in yellow), 30–60 µm (in red), 60–90 µm (in blue), 90–120 µm (in green). And then, the re‐segmented labels were reconstructed three‐dimensionally to show the aggregation extent of steatotic hepatocytes with the distances changing to the central veins. From Figure 7M,N, and Movie S1 (Supporting Information), the central veins were reconstructed in white color, and the steatotic regions of different distances in different colors were displayed three‐dimensionally. By a quantitative analysis of these steatotic regions, we found that the steatotic regions in yellow, red, blue, and green color accounted for 68%, 56%, 30%, and 18% in volume, respectively (**Table** **3**). To clarify the changes in the volume of steatotic regions surrounding veins of different diameters, we also extracted the steatotic regions within a 150‐micrometer range from the surface of individual central veins and conducted a quantitative analysis (Figure S9, Supporting Information). The results showed that the volume of steatotic regions surrounding central veins of different diameters did not change with the diameter of the blood vessels (**Table** **4**). This phenomenon revealed that the closer to the central vein, the higher the proportion of steatotic hepatocytes.

**Table 3:** The volume of the labeled steatotic regions at different distances with different colors from the surface of central veins in Figure 7L,M.

Object	Distance from CV [µm]	Volume3D [10^8^ µm^3^]	Percentage [%]
Region Volume	0–30	3.32	/
	30–60	5.47	/
	60–90	7.34	/
	90–120	8.52	/
Steatotic Region Volume	0–30 (yellow)	2.26	68
	30–60 (red)	3.05	56
	60–90 (blue)	2.22	30
	90–120 (green)	1.56	18

Note: Data are based on measurements from the labels of steatotic hepatocytes in different colors in Figure 7L,M.

**Table 4:** The volume of the labeled steatotic regions around central veins of different diameters in Figure S8 (Supporting Information).

CV Diameter [µm]	Region volume around CV [10^7^ µm^3^]	Steatotic Region Volume [10^7^ µm^3^]	Percentage[%]
>150	6.19	2.83	46
100‐150	7.39	3.33	45
<100	4.83	2.36	49

## 3 Discussion and Conclusion

In this study, we have utilized the MOST imaging and Nissl staining method to achieve the first high‐precision whole mouse liver atlas at a resolution level of 0.35 µm × 0.35 µm × 2 µm, which demonstrated the capability for visualization and analysis of the entire mouse liver. Taking advantage of this method, we investigated the pathological dataset of whole mouse liver, indicating that there are no evident pathological differences among the different liver lobes. The potential interlobar distinctions may require further in‐depth research or more innovative analytical methods to be fully elucidated. We then constructed a detailed 3D pathological mapping of the left liver lobe in the CCl_4_‐induced liver fibrosis mice at a resolution level of 0.35 µm × 0.35 µm × 1 µm, and provided the clear visualization and elaborate analysis of multiple structures in the mouse liver.

With technological advancements, increasingly large‐volume imaging techniques are being applied to liver imaging. The use of CT and MRI for noninvasively visualizing complete hepatic vessels has greatly enhanced the clinical identification of liver conditions. These imaging modalities were also employed for small animal imaging to precisely identify and describe pathological processes of the liver.^[^ ^20^ ^]^ To break through the limitation in the resolution of CT and MRI techniques, the synchrotron radiation micro‐computed tomography (SR‐µCT) enable scientists to identify the hepatic sinusoids three‐dimensionally in normal and CCl_4_‐injured mouse liver.^[^ ^14^ ^]^ The other work adopting PCI‐CT (phase‐contrast imaging computed tomography) could assess the morphological changes in liver microvasculature that result from fibrosis and allow characterization of the anatomical and pathological features of the microvasculature.^[^ ^21^ ^]^ Although these imaging methods have achieved a relatively high resolution, the scanning range is typically limited.

To balance the detection range and imaging resolution simultaneously, the combination of light sheet microscopy and tissue clearing method facilitates the visualization of large, or even intact liver structures. The light‐sheet imaging and whole‐mount immunostaining (iDISCO) were performed to obtain 3D sympathetic innervation in the liver, and explore the integrity of the hepatic nervous system in nonalcoholic fatty liver disease.^[^ ^16^ ^]^ The other study developed a more effective clearing method for light‐sheet imaging and performed a systematic and integrated analysis of the hepatic sinusoid.^[^ ^22^ ^]^ These imaging modalities make it more easily to achieve visualization of target liver structures when coupled with the appropriate labeling strategy compared with MOST technology. However, in the practical experimental procedure, the effect of tissue transparency significantly impacted the imaging quality. It is noteworthy that a method named CODA was introduced to reconstruct exceptionally large tissues at subcellular resolution using serially sectioned hematoxylin and eosin‐stained tissue sections.^[^ ^23^ ^]^ As a bright‐field 3D imaging method, the CODA requires manual additional registration. However, one of the advantages of MOST is that no additional registration is needed because of the accurate spatial positioning of the images. Most mechanical slicing methods suffer from serious deformation, which can be overcome using MOST.

Here, we employed the advanced bright‐field MOST technology and liver Nissl staining to obtain the simultaneous visualization of central veins, portal veins, arteries, bile ducts, hepatic sinusoids, and hepatic cells at sub‐micron resolution in the intact liver. The acquisition of images at sub‐micron resolution for liver lobes or whole livers indeed generates an enormous number of datasets. The continuous data acquisition of the whole liver was acquired at a thickness of 2 µm and still lasted for more than 20 days, producing more than 9000 coronal sections and occupying ≈1 terabyte in the JPEG format. And the left liver lobes, which were all sectioned with a voxel size of 0.35 µm × 0.35 µm × 1 µm, yielded over 700 gigabytes dataset from ≈10 000 coronal sections. However, there is currently no efficient integrated software or algorithm for processing and analyzing these massive bright‐field images. The 3D reconstruction of the Terabyte‐level data generated by sub‐micron level imaging across the entire mouse liver is severely constrained by the configuration of computer hardware and software.^[^ ^24^ ^]^ Both 3D high‐precision rendering and quantitative analysis face numerous challenges, especially quantitative analysis, which is more dependent on high‐performance computer configurations and consumes more time and effort. Conventional laboratory computers and software are simply unable to handle such large data. In addition to the high demands on computer hardware, there is still a lack of effective fully automated tools for high‐throughput image data processing.^[^ ^25^ ^]^ A single algorithmic breakthrough cannot solve all the main challenges, and the development of high‐performance computer equipment and efficient image processing software or algorithms remains a long way off. Optimizing efficient data processing methods through human‐computer interaction using technologies such as artificial intelligence is expected to play a more important role in the near future.^[^ ^26^ ^]^

Our study suggested several benefits of the application of bright‐field 3D imaging. For example, the texture and morphology of 3D structures reconstructed from bright‐field images are more realistic, unlike fluorescent 3D images which are often a blurred representation of the actual object under the microscope. This bright‐field 3D imaging modality has helped us to yield a liver structural atlas that more accurately represents the actual condition. Actually, compared with our data, the liver vessels visualized from the fMOST‐generated dataset were quite small and abnormal.^[^ ^17^ ^]^ Our processing method for the high‐throughput bright‐field images provides an alternative non‐fluorescent labeling and imaging method for visualizing multiple structural components in liver research and elevates pathological research from a 2D to a 3D perspective. Previous studies utilizing fluorescent labeling and imaging techniques^[^ ^15^, ^16^, ^27^ ^]^ have greatly facilitated comprehensive visualization of specific fine structures. Nevertheless, these studies encounter challenges when attempting to capture multiple structures simultaneously, including various tubular and linear architectures, as well as cellular components. Consequently, we advocate selecting an appropriate approach that meet the specific imaging requirements. For the 3D visualization of tiny and specific structures such as nerves, a fluorescence‐based imaging strategy emerges as the most effective option. Meanwhile, for the high‐precision 3D reconstruction of multiple structures, the technology of MOST and Nissl staining are recommended for their superior capabilities.

We found that the surface nodularity was quite prominent, and the primary changes of vascular network occurred in the central veins with significant decline of mean diameter and increase of length density in the CCl_4_‐induced hepatic fibrosis, which suggested the smaller vessels increased due to the hepatic sinusoidal capillarization. The quantitative analysis of the hepatic sinusoids showed that the mean diameter and volume fraction significantly decreased, and the length density and number of segments remarkably increased. Besides, the steatotic hepatocytes and damaged sinusoids also surrounded the central veins rather than the portal veins. We speculated that these region‐specific distributions were caused by the cytochrome P450 enzymes, of which the expression levels are known to be higher in the centrilobular area than in the periportal region.^[^ ^28^ ^]^ Meanwhile, the CCl4 is primarily metabolized by cytochrome P450 enzymes to a toxic metabolite.^[^ ^29^ ^]^ Thus, the hepatocytes around the central veins suffered the most severe damage, which had been verified by the quantification of the steatotic regions of different distances from the surface of central vein. We found that the degree of steatosis was positively correlated with the proximity to the central vein, but was not significantly related to the diameter of the blood vessels. Additionally, the degree of steatosis also showed little variation among the different liver lobes, as revealed by the investigation of whole‐liver steatotic regions. These results revealed that the pericentral sinusoids, particularly close to the central veins, were severely damaged in the volume and tend to be more complex in the hepatic fibrosis. The in‐depth analysis using deep learning model revealed that the proportion of deformed hepatocytes volume reaches 30.76%. According to the Masson's trichrome staining, the regions of collagen deposition overlapped with the regions of hepatocyte steatosis. Considering the relationship between collagen deposition and liver fibrosis, we speculate that the proportion of steatotic regions may reflect the degree of fibrosis to some extent. These findings precisely reveal a spatial complexity in liver pathology from a 3D perspective.

It should be noted that there are several shortcomings in this study that are worth discussing. First, in view of the similar pathological characteristics among the liver lobes (Figure S4, Supporting Information and Table 2), and to minimize the imaging time and the workload of image analysis, this study conducted in‐depth pathological analysis on the left lobe of the liver. A more comprehensive and in‐depth study across all liver lobes will be conducted in subsequent investigations. Second, compared with tracing fine lumen/line structures in fluorescent specific‐labeling image sequence, extracting these structures from massive bright‐field image data is extremely challenging and time‐consuming. All of these lumen/line structures in the Nissl staining dataset were identified according to the differences in the anatomical distribution and endothelial cell morphology. But unlike the automatic segmentation of the central vein and portal vein based on the connectivity and region growing algorithm, it is particularly difficult to segment the artery and bile duct, as well as the peribiliary vascular plexus and lymphatic vessel automatically. The simplest and most efficient solution is to manually use the Magic Wand tool to extract these structures with poor continuity. Due to the huge workload, we thus segmented the peribiliary vascular plexus and lymphatic vessels in the local area rather than over the entire liver lobe. Third, using fluorescence 3D imaging to study characteristic structures with tiny diameters such as nerve fibers might be more convenient than using the MOST system. Fluorescence images typically have homogeneous signals and backgrounds, making them particularly suitable for the 3D reconstruction of complex structures that are difficult to trace and extract automatically.

Our work comprehensively and systematically reveals the 3D pathology of the liver fibrosis, and shows that the CCl_4_‐induced liver injury comprising vascular changes, sinusoidal deformation, hepatic steatosis, and collagen deposition was region‐specific and central vein‐associated. Although further study is necessary to clarify the physiological role and mechanism underlying the notable structural alteration in liver fibrosis, our research yielded a cross‐scale high‐precision panorama of multiple liver structures associated with fibrosis in a single sample. This will facilitate a better understanding of the hepatic anatomical features under the pathological state of fibrosis, and exhibit extensive application prospects in the pathological assessment of other tissues from organ‐wide level.

## 4 Experimental Section

### CCl_4_ Administration and Sample Preparation

The 2‐month‐old male C57BL/6 mice were provided in this work and purchased from Shanghai SLAC Laboratory Animal Co, Ltd (Shanghai, China). The mice were randomly divided into two groups (*n*  =  10 in each group) as follows: oil group, CCl4‐treated model group. The CCl_4_‐treated mice were injected intraperitoneally with 10% CCl_4_ (10 mL kg^−1^ of body weight) diluted in corn oil twice a week for 6 weeks. The mice in the oil group were injected with corn oil alone (*n* = 10). After 6 weeks administration, the mice were fasted overnight and anesthetized after weighing. Blood was collected via the orbital venous sinus under anesthesia. All animal procedures were performed in accordance with the National Institutes of Health Guide for the Care and Use of Laboratory Animals, under protocols approved by and strictly following the guidelines of the Institutional Animal Care and Use Committee (IACUC, No. 2020‐02‐GZB‐05).

For liver function examination, ALT and AST (*n* = 8) were measured with biochemical kits purchased from Nanjing Jiancheng Biological Engineering Research Institute (Nanjing, China). 1) Centrifuge the blood samples for 10 min at 1000 rpm to separate the serum from the cellular components, and then carefully aspirate the supernatant and transfer it to a clean tube. 2) Prepare the reagents according to the manufacturer's instructions provided with the Nanjing Jiancheng Bioengineering Institute's kit. 3) Add the serum samples to the reaction mixture containing the substrates for ALT and AST. 4) Initiate the enzymatic reaction by incubating at 37 °C for 20 min. 5) Gently agitate the microplate to mix the contents, let it stand at room temperature for 15 min, measure the OD values of each well at a wavelength of 505 nm using a microplate reader. 6) Calculate the absolute OD values (subtracting the OD value of the control well from the OD value of the test well) and use the standard curve to determine the corresponding ALT/GPT activity values.

Before cardiac perfusion, the mice were fasted overnight and then anesthetized with an intraperitoneal injection of Zoletil 50. The mice were subsequently perfused with 0.01 m PBS (Yeasen Biology) followed by paraformaldehyde (Ribiology, R1001). After perfusion, the intact livers were carefully extracted from the abdomen and postfixed in 4% paraformaldehyde for 24 h at room temperature.A whole liver from 2‐month‐old male C57BL/6 mouse, a whole liver of CCl_4_ model mice and the largest left liver lobes of CCl_4_‐administrated mice as well as oil‐administrated mice were selected for whole‐mount Nissl staining. The left lobes of experimental mice liver were also conducted H&E and Masson's trichrome staining, which was stained and scanned by Servicebio company (Wuhan, China). The whole‐mount Nissl staining method followed the previous description.^[^ ^19b^ ^]^ Briefly, the whole livers and left livers (*n* = 5) were dyed for at least 12 days using a 2.5% thionine (Sigma–Aldrich, 861340) solution. Then, the dyed livers were subsequently dehydrated with a graded series of ethanol and acetone solutions. Next, the dehydrated livers were immersed in Spurr resin (Sigma–Aldrich, EM0300), and then the livers were embedded and placed in 60 °C oven polymerizing for 2 days.

The resin‐embedded specimens of left liver lobes were subsequently imaged with the MOST system (Wuhan OE‐Bio Co., Ltd, Wuhan, China) to acquire raw datasets with a voxel size of 0.35 µm × 0.35 µm × 1 µm. The whole mouse liver was sectioned at a thickness of 2 µm to economize acquisition time. It still took more than 20 days to collect the complete raw data. The MOST system^[^ ^30^ ^]^ employs bright‐field reflection imaging, relying on mechanical sectioning with a diamond knife to achieve imaging depth. During the sectioning process, imaging is performed simultaneously, and the sample information is acquired using a line‐scanning and strip‐stitching method.

### Image Preprocessing and Optimization

The raw images were preprocessed for brightness nonuniformity correction and seamless image stitching to achieve coronal image sequence based on a C++ program.^[^ ^31^ ^]^ Subsequently, an image optimization method was conducted to further improve background uniformity, periodic noise, and contrast of the processed coronal images. The procedures including background correction, noise reduction, and contrast enhancement were implemented to optimize the coronal images.^[^ ^32^ ^]^

### Visual Reconstruction of Multiple Liver Structures

The optimized high‐quality coronal images were resampled to 1 µm × 1 µm × 1 µm and used to extract different liver structural information in different gray levels. All of liver structures were rendered and extracted according to previous method^[^ ^19b^ ^]^ using Amira 3D software (2022.2). The different lobes and gall bladder of the whole liver were interactively segmented using Segmentation editor in Amira.

### 1) Segmentation and Quantification of Central Veins and Portal Veins

The tubular structures such as the central veins, portal veins were segmented using the Segmentation editor in Amira based on the region growing algorithm. Then, the label data was calculated using the Label Analysis module, and removed from the input label analysis labels whose measure does not fulfill a filter formula using the Analysis Filter module. The filtered label data was computed by Auto Skeleton module to obtain morphological parameters including mean diameter, number of segments, total length, and total volume (*n* = 3 liver lobes from 3 mice per group). The volume of the liver lobe was obtained by computing the label images of the liver lobe using the Label Analysis module (*n* = 3 liver lobes from 3 mice per group). The length density was calculated by dividing the total length of the vessels by the volume of the liver lobe (meters per cubic millimeters, n = 3 liver lobes from 3 mice per group). The volume fraction was calculated by dividing the total volume of the vessels by the volume of the liver lobe (*n* = 3 liver lobes from 3 mice per group).

### 2) Segmentation of Arteries, Bile Ducts, Peribiliary Vascular Plexus, and Lymphatic Vessels

The smaller tubular structures including the arteries and bile ducts were relatively difficult to automatically segment. The arteries and bile ducts on the basis of the differences in the morphology of their endothelial cells was identified, and manually segmented them using Amira. The peribiliary vascular plexus and lymphatic vessels of the local region were also manually segmented using Magic wand tool in the Segmentation Editor of Amira.

### 3) Segmentation and Quantification of Hepatic Sinusoids

The hepatic sinusoids were rendered and segmented using 30‐µm‐thick coronal slices at a voxel size of 0.35 µm × 0.35 µm × 1 µm from both the oil and CCl_4_ groups. The hepatic sinusoids together with vessels were segmented using the Interactive Thresholding module (Figure 5E,F), and then the vessels were subtracted using the Magic Wand tool, retaining only the liver sinusoids (Figure 5G,H). The morphological parameters were concerning mean diameter, volume fraction, length density, and number of segments were calculated using the ROIs (*n* = 3 ROIs from 3 mice per group) shown in the Figure 5G,H. The data blocks (oil: 3211 × 2825 × 2648 µm; CCl_4_: 2906 × 2878 × 2484 µm) as shown in Figure 6A,B at the edge of the liver lobes were also cropped and rendered to display the spatial relationship among the central veins, portal veins, hepatic sinusoids, and steatotic hepatocyte.

### Automatic Cell Segmentation Using Deep Learning Model in Arivis

The steatotic hepatocytes of the data block and the whole liver were segmented automatically using deep learning models for image segmentation in ZEISS Arivis software. The user‐friendly interface in ZEISS arivis Cloud makes it easy to train your own deep learning models for image segmentation, without the need for coding. The detailed segmentation procedures were as follows:

1) Automatic Cell segmentation

**Upload training data**: a training data block form a CCl_4_‐treated mouse was selected and uploaded it to the Arivis cloud.

**Annotation of target and background objects**: A trained individual used the brush tool to draw the target objects and background objects until obtaining a sufficient amount of data for training. This step was crucial for creating the ground truth data for training the model. Next, semantic segmentation was selected for model training.

**Training the Model**: Once you have enough annotated images, initiate the training process by clicking the “Train” button. This will teach the deep learning model to recognize and differentiate the various object classes.

**Model Evaluation**: After training, the evaluation of model performance can be obtained to ensure it can accurately segment the objects of interest. As shown in Figure S7 (Supporting Information), three training curves including loss, accuracy, and intersection over union (IoU) were generated after the model training was successfully completed. Each plot displays the curves for both the training data (in yellow) and the validation data (in blue). After ≈3500 training iterations, the loss curve indicates that the loss function for the training data was consistent with the estimated loss function, both showing a downward trend. The accuracy curve reveals that the training accuracy rises gradually to the validation accuracy and exceeds 99%. It can be observed from the curve that for both the training and validation, IoU values are on an upward trend, both approaching 90%.

**Using the Trained Model**: Once the model was trained and evaluated, you can use it for segmentation by opening it in a pipeline. The trained model can then be applied to new images for automatic segmentation.

**Segmentation Operation**: With the trained model in the pipeline, use the segmentation operation to process your images and generate segmentation masks for the annotated objects.

**Post‐Processing**: After obtaining the initial segmentation results, you may perform additional steps such as object classification, filtering, or refinement to improve the accuracy of the segmentation.

**Analysis and Export**: Finally, analyze the segmented data as needed, and export the results, such as object features or segmentation masks, for further use or documentation.

2) Visualization and quantification of segmented steatotic hepatocytes

The grayscale images of steatotic hepatocytes were achieved multiplying the segmented data by original coronal data. The segmented steatotic hepatocytes were reconstructed in Amira. The volume of steatotic hepatocytes was calculated using Label Analysis module in Amira. The quantitative analysis of the interlobar differences

The re‐segmentation and quantification of the steatotic regions at different distances including 0–30 µm (in yellow), 30–60 µm (in red), 60–90 µm (in blue), 90–120 µm (in green) from the central veins was performed using Imaris 10.1.0. The distances were defined as the distances from the surface of the central veins.

3) Quantitative analysis of steatotic hepatocytes surrounding individual CV branch

The individual branch of central vein was extracted from the label data as shown in Figure 6B. The label data for the surrounding regions was obtained by dilating 150 micrometers from the surface of the central veins (Figure S8C, Supporting Information). The label data of the steatotic hepatocytes was obtained by multiplying the 150‐µm‐radius label data and the label segmented using deep learning model (Figure S8D, Supporting Information). The grayscale images of the steatotic regions surrounding the central vein were achieved by multiplying the label of the steatotic regions and the raw grayscale images (Figure S8E, Supporting Information). The quantitative analysis of the volume of the steatotic regions surrounding the different diameters of central veins was conducted using Imaris 10.1.0.

### Statistical Analysis

All data were analyzed in GraphPad Prism 9. A two‐tailed Student's *t*‐test was employed to assess statistical significance in values between the oil and CCl_4_ group. Data were presented as mean ± s.e.m. A P value < 0.05 was considered statistically significant.

## Conflict of Interest

The authors declare no conflict of interest.

## Author Contributions

X.Z. and W.Y. contributed equally to this work. X.Z., X.Y., and Z.G. conceived and designed the research. X.Z. and W.Y. performed sample preparation, and data acquisition. X.Y. performed image optimization. X.Z. performed image preprocessing, visualization and quantitative analysis. W.Y. performed manual segmentation. X.L. and Y.Z. performed AI segmentation. W.Y., S.L., and Y.Z. performed animal experiments and liver function assay. All authors analyzed the data. X.Z., X.Y., Z.G., and W.Y. wrote the manuscript.

## Supporting information



Supporting Information

Supplemental Movie 1

## Data Availability

The data that support the findings of this study are available in the supplementary material of this article.
