# Supplementary material for: Generation of a High‐Precision Whole Liver Panorama and Cross‐Scale 3D Pathological Analysis for Hepatic Fibrosis
Source: Adv Sci (Weinh). 2025 Mar 24;12(19):2502744. doi: 10.1002/advs.202502744 (PMC12097011; doi:10.1002/advs.202502744)
Supplement: Supplementary file 1 — Supporting Information [file ADVS-12-2502744-s002.docx]

# Supporting Information

Title

Generation of a high-precision whole liver panorama and cross-scale 3D pathological analysis for hepatic fibrosis

*Xiaochuan Zhang^#,*^, Weicheng Yang^,#^, Xiaoliang Li, Yanli Zhao, Zongneng Xie, Shuangqu Li, Yue Zeng, Xiaoxu Hao, Xiaohong Xin, Yu Zhang, Zixuan Feng, Hualiang Jiang^†^, Zhaobing Gao^,*^, Xianzhen Yin^*^*

Figure S1-S9

Movie S1, related to Figure7L

**
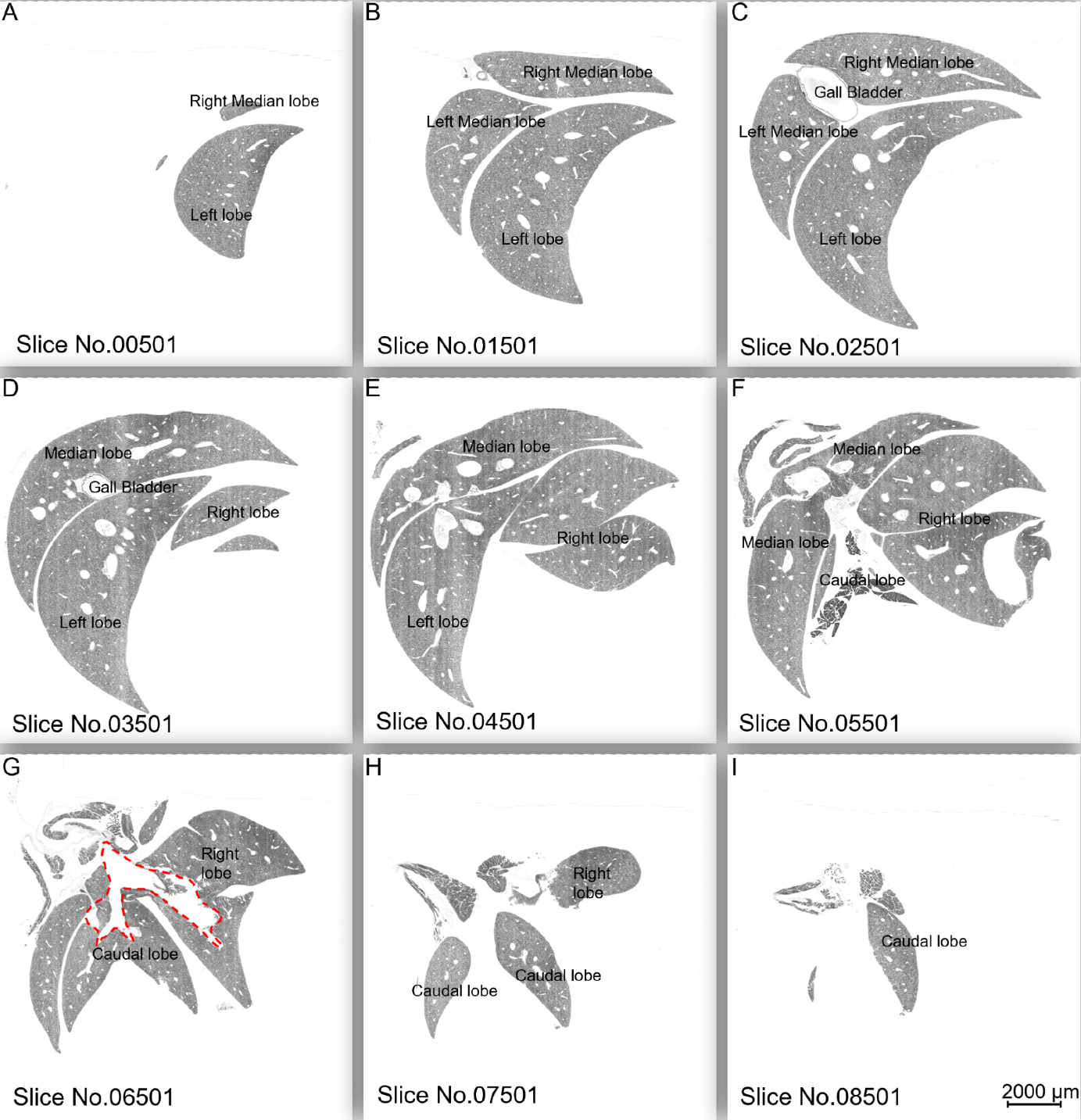
Figure S1 Representative images of coronal section from a 2-month-old male C57BL/6 mouse**

**(A-I) Representative serial liver sections from the the liver apex to hilus. The dotted line in G denotes the liver hilus.** Scale bar, 2000 μm.


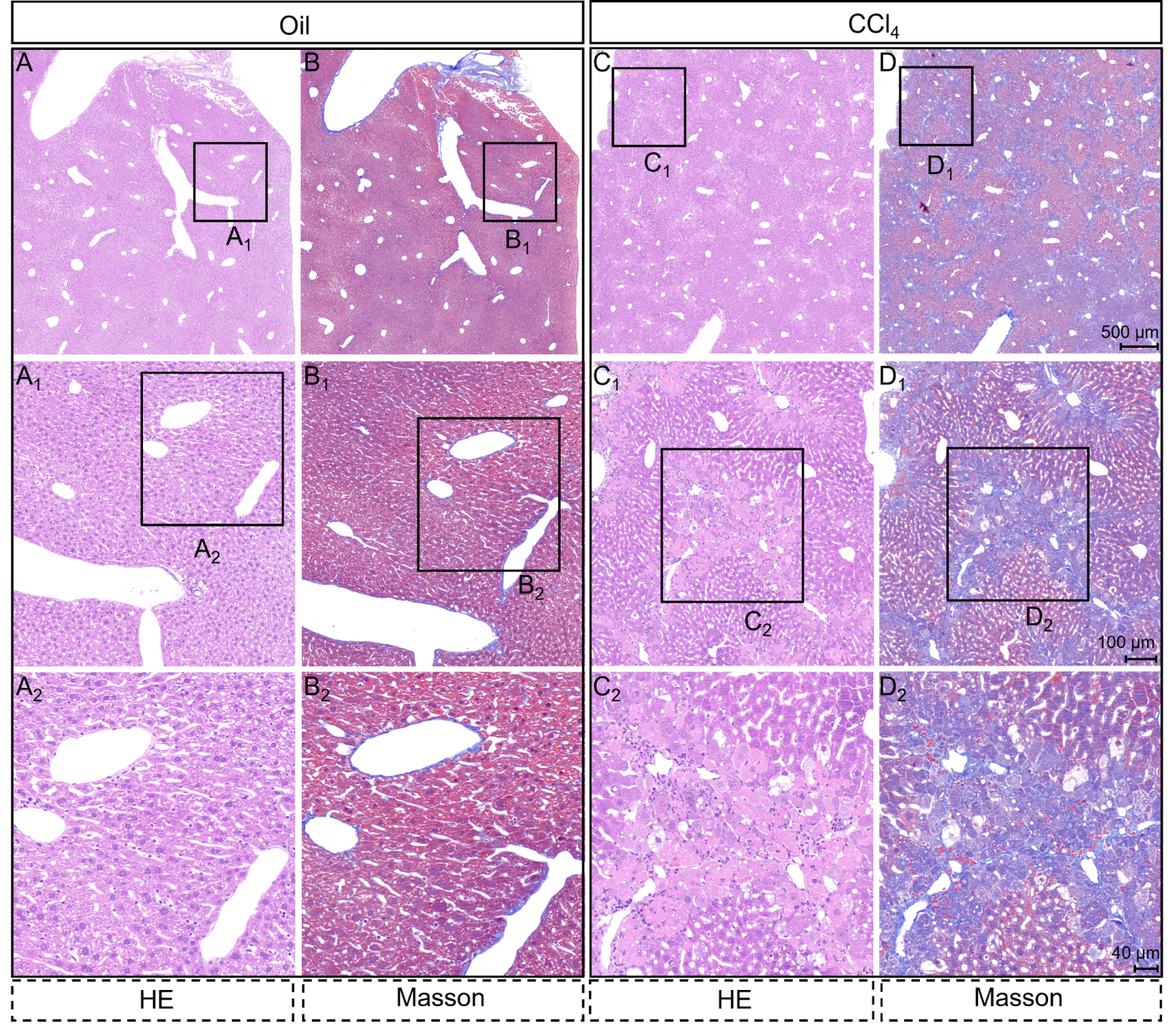


**Figure S2 Two-dimensional histological assessments of liver fibrosis from the left lobes of oil and CCl_4_ group**

(**A, B**) Representative liver images of H&E and Masson’s trichrome staining from the left lobes of the oil group. (**C, D**) Representative liver images of H&E and Masson’s trichrome staining from the left lobes of the CCl_4_ group. Enlarged views of the boxes in A-D were shown in A_1_-D_1_. Enlarged views of the boxes in A_1_-D_1_ were shown in A_2_-D_2_. Scale bar in A-D, 500 μm. Scale bar in A_1_-D_1_, 100 μm. Scale bar in A_2_-D_2_, 40 μm.


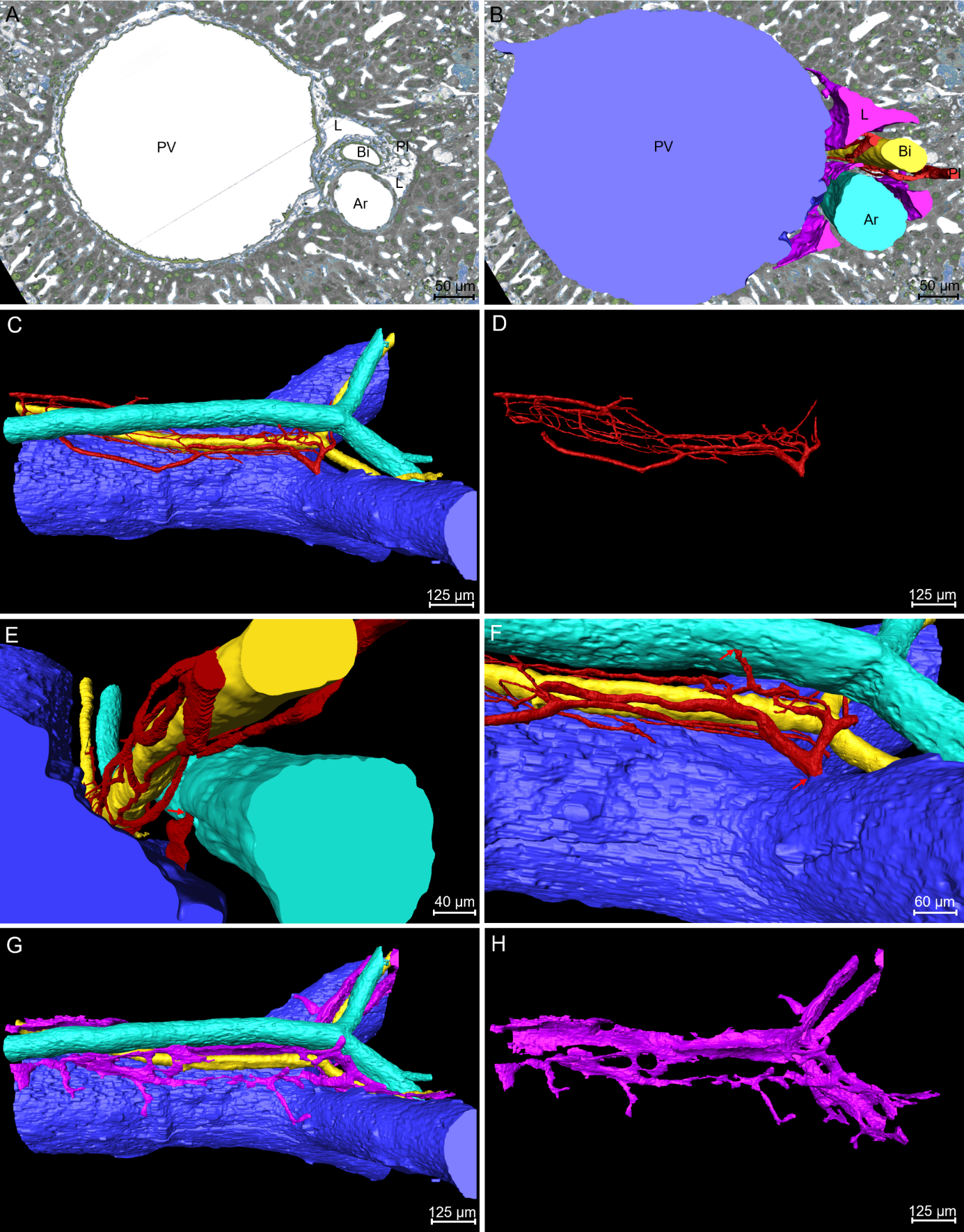
**Figure S3 Segmentation of the portal triad in local region**

(**A, B**) Representative coronal image of portal triad and the segmented structures. Portal vein, PV, in blue; artery, Ar, in cyan; bile duct, Bi, in yellow; peribiliary vascular plexus, Pl, in deep red; and lymphatic vessel, L, in purple. Scale bar in A, B, 50 μm. (**C-F**) Simultaneous visualization of portal vein, artery, bile duct, and peribiliary vascular plexus. The arrow denoted the connection points. Scale bar in C,D, 125 μm. Scale bar in E, 40 μm. Scale bar in F, 60 μm. (**G-H**) Simultaneous visualization of portal vein, artery, bile duct, and lymphatic vessel. Scale
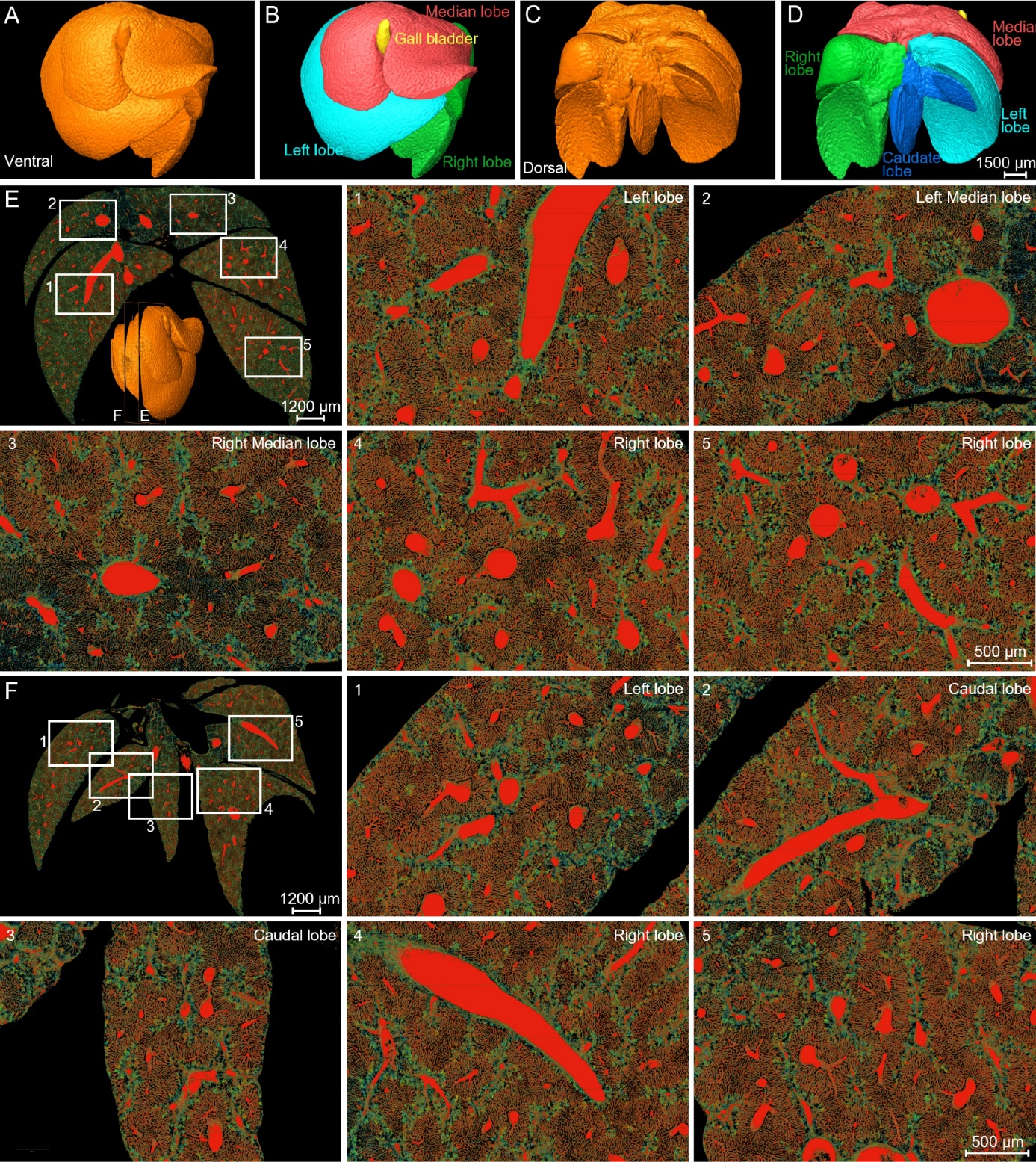
bar in G, H, 125 μm.

**Figure S4 Surface reconstruction of whole liver and volume rendering of representative coronal slices from CCl_4_ mice**

(**A-D**) Surface reconstruction and liver lobes segmentation of the whole mouse liver from the CCl4 mouse. (A,B) Ventral view. (**C-D**) Dorsal view. Scale bar in A-D, 1500 μm (**E, F**) Representative liver images of coronal slices using volume rendering from the CCl_4_ mouse. The orthogonal slices denoted the position of the coronal slices in E and F. Scale bar, 1200 μm. Enlarged views of the boxes in E and F were shown in E_1-5_ and F_1-5_, respectively. Scale bar in E_1-5_, 500 μm. Scale bar in F_1-5_, 500 μm.


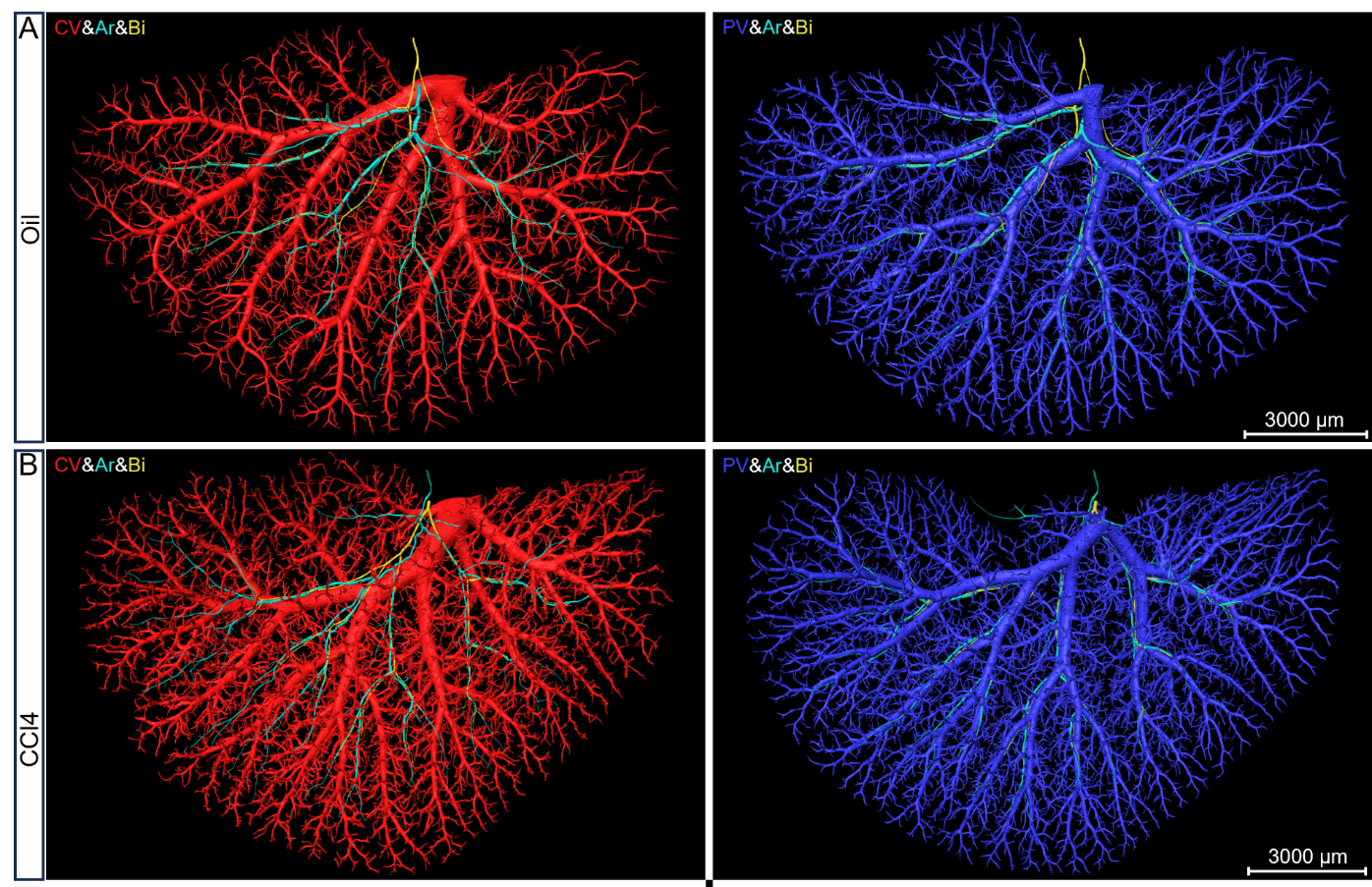


**Figure S5 Dorsal view of the reconstructed tubular structures showing the spatial relationship among central vein (CV), portal vein (PV), artery (Ar), and bile duct (Bi) in the oil and CCl_4_ group**

(**A**) Oil group. Scale bar, 3000 μm. (**B**) CCl_4_ group. Scale bar, 3000 μm.


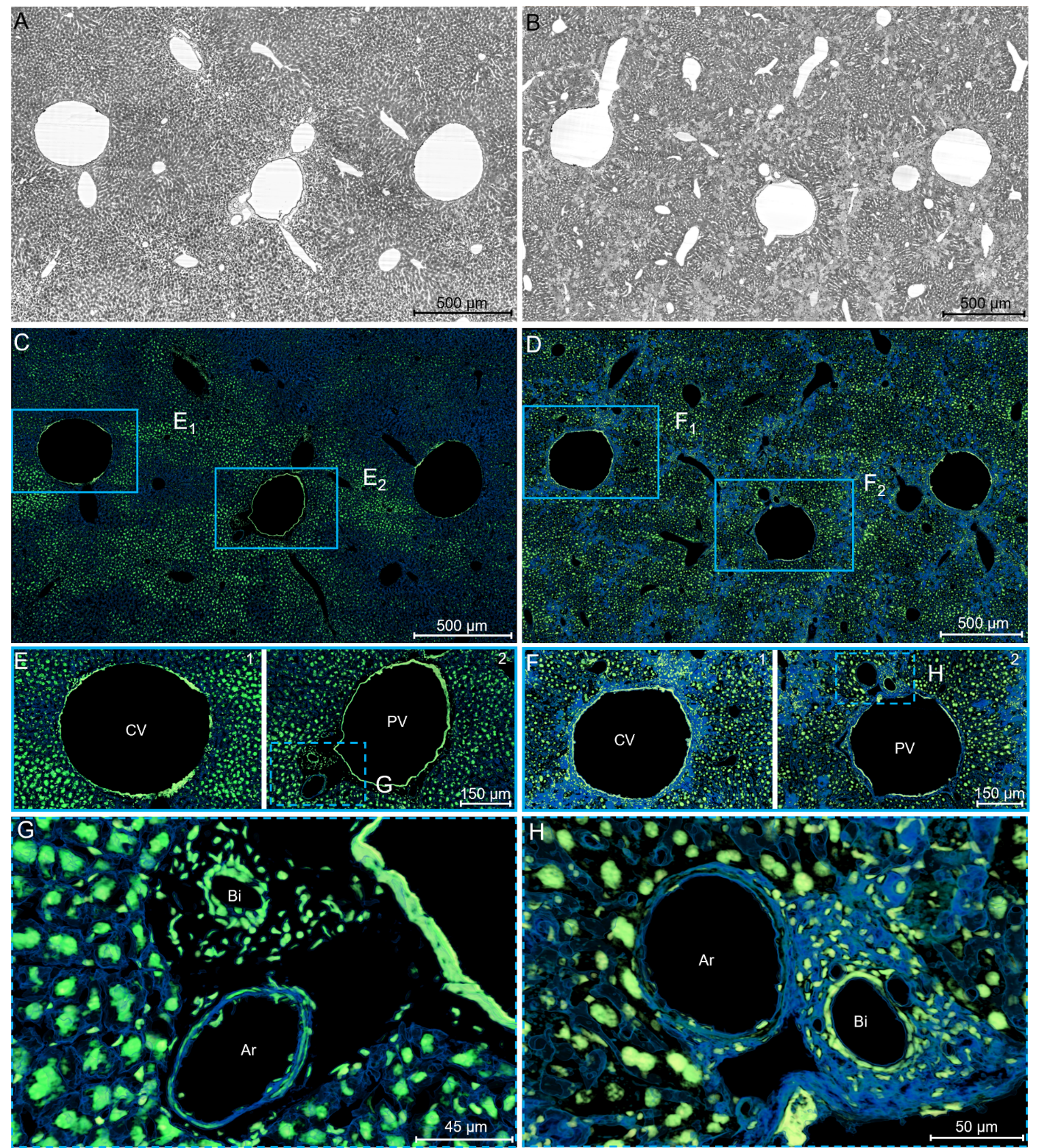
**Figure S6 Visualization of cells in** **representative coronal slices**

(**A, B**) Representative images of the same coronal section as shown in Figure 5 of both the oil and CCl_4_ group. Scale bar, 500 μm. (**C, D**) Visualization of the cells in the both oil and CCl_4_ group, showing normal cell arrangement in the oil group and sparse cell distribution in the CCl_4_ group. The blue color represents the cytoplasm, and the green color represents cell nucleus. The cytoplasm of the steatotic hepatocyte was enlarged, resulting in more pronounced blueness around the central vein. Scale bar, 500 μm. (**E, F**) Enlarged views of the boxes in C-D, showing the cells reconstruction around the central vein and portal vein. Scale bar in E_1-2_, F_1-2._, 150 μm.(**G, H**) Enlarged views of the boxes in E-F, illustrating the differences in the endothelial morphology between the artery (Ar) and bile duct (Bi). Scale bar in G, 45 μm. Scale bar in H, 50 μm.


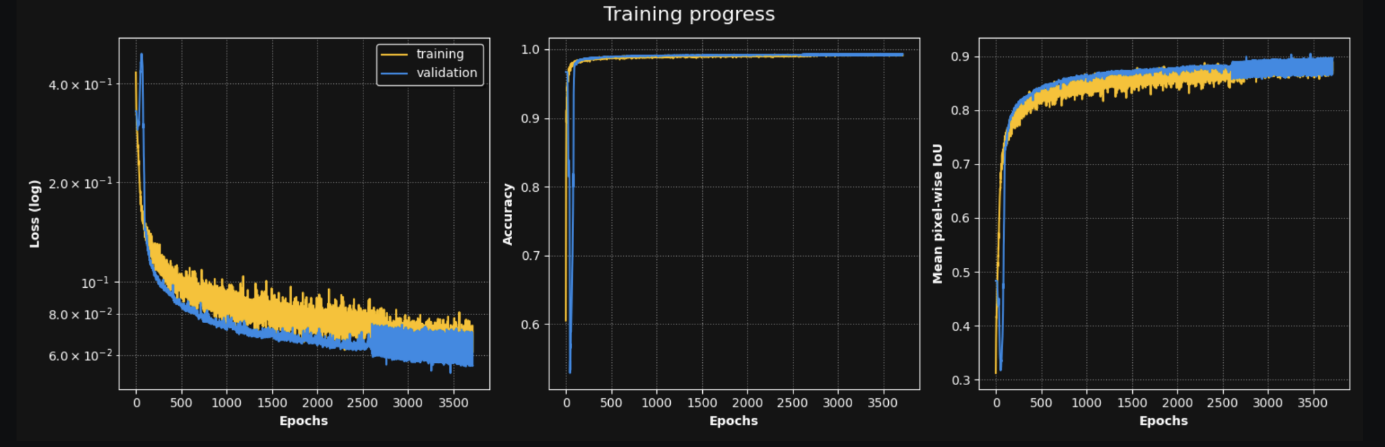


**Figure S7 Model evaluation curve including loss, accuracy, and intersection over union**

Each plot shows curves for the training data (yellow) and validation data (blue) respectively. Loss(left panel): Loss is the penalty for a bad prediction. It is a number indicating how bad the model‘s prediction was on a single example. If the model’s prediction is perfect, the loss is zero; otherwise, the loss is greater. The goal of deep learning algorithm is to minimize loss. Accuracy(middle panel): Accuracy is a metric used during training to evaluate the model. It represents the fraction of accurate predictions that the model made. Intersection over Union (IoU, right panel): IoU is the most popular metric to evaluate the quality of semantic segmentation. It is a measurement to quantify the accuracy of overlap between two areas.


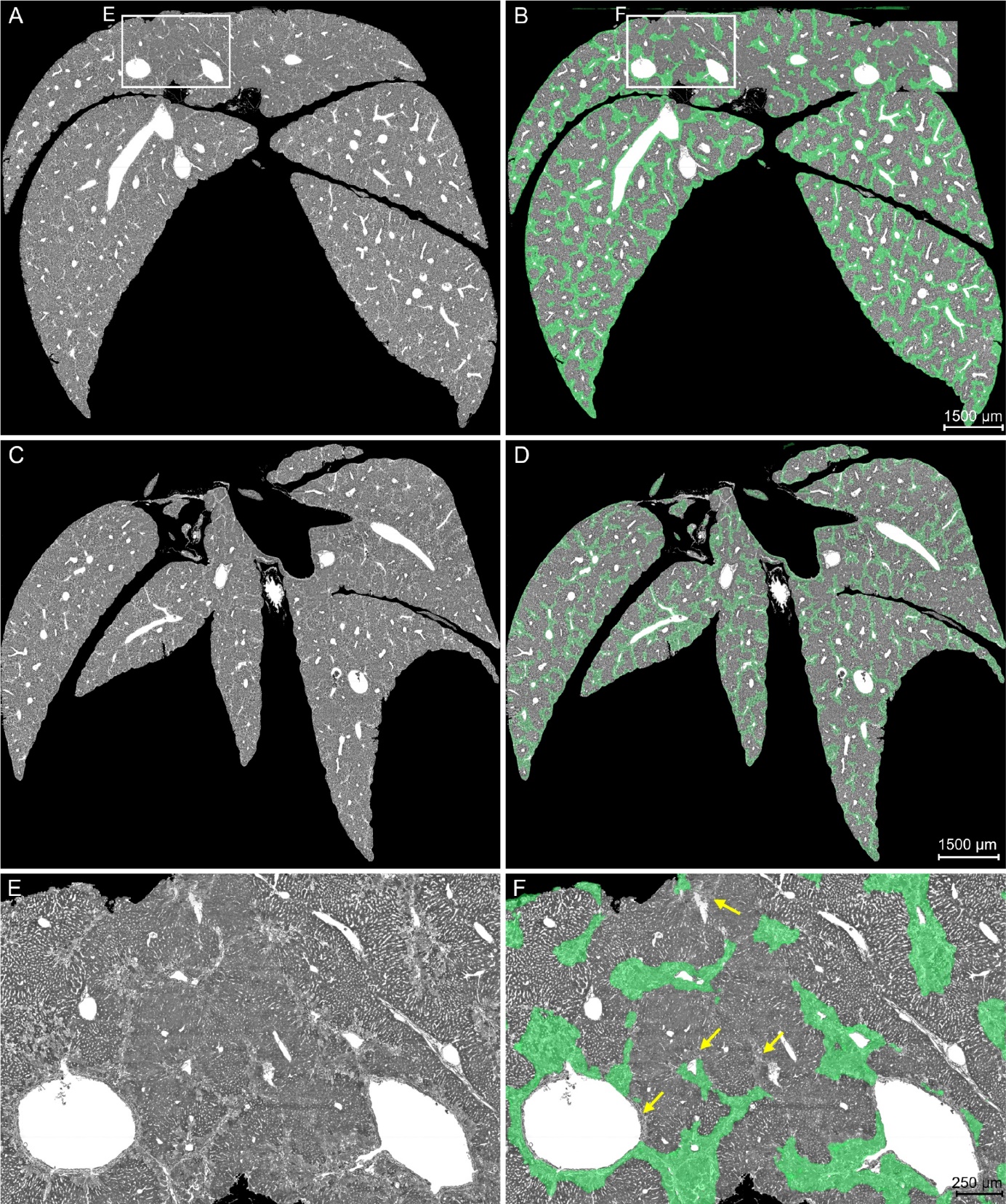


**Figure S8 Segmentation and quantitative analysis of the** **steatotic regions in different liver lobes**

(**A-D**) Representative images of coronal section showing the identification and segmentation of steatotic regions in the whole liver. The coronal sections in panels A and C correspond to those depicted in Figure S4E and Figure S4F, respectively. Enlarged views of the boxes in A and B were shown in E and F. The yellow arrows denoted the steatotic regions that were not identified and segmented accurately. Scale bar in A-D, 1500 μm. Scale bar in E and F, 250μm.


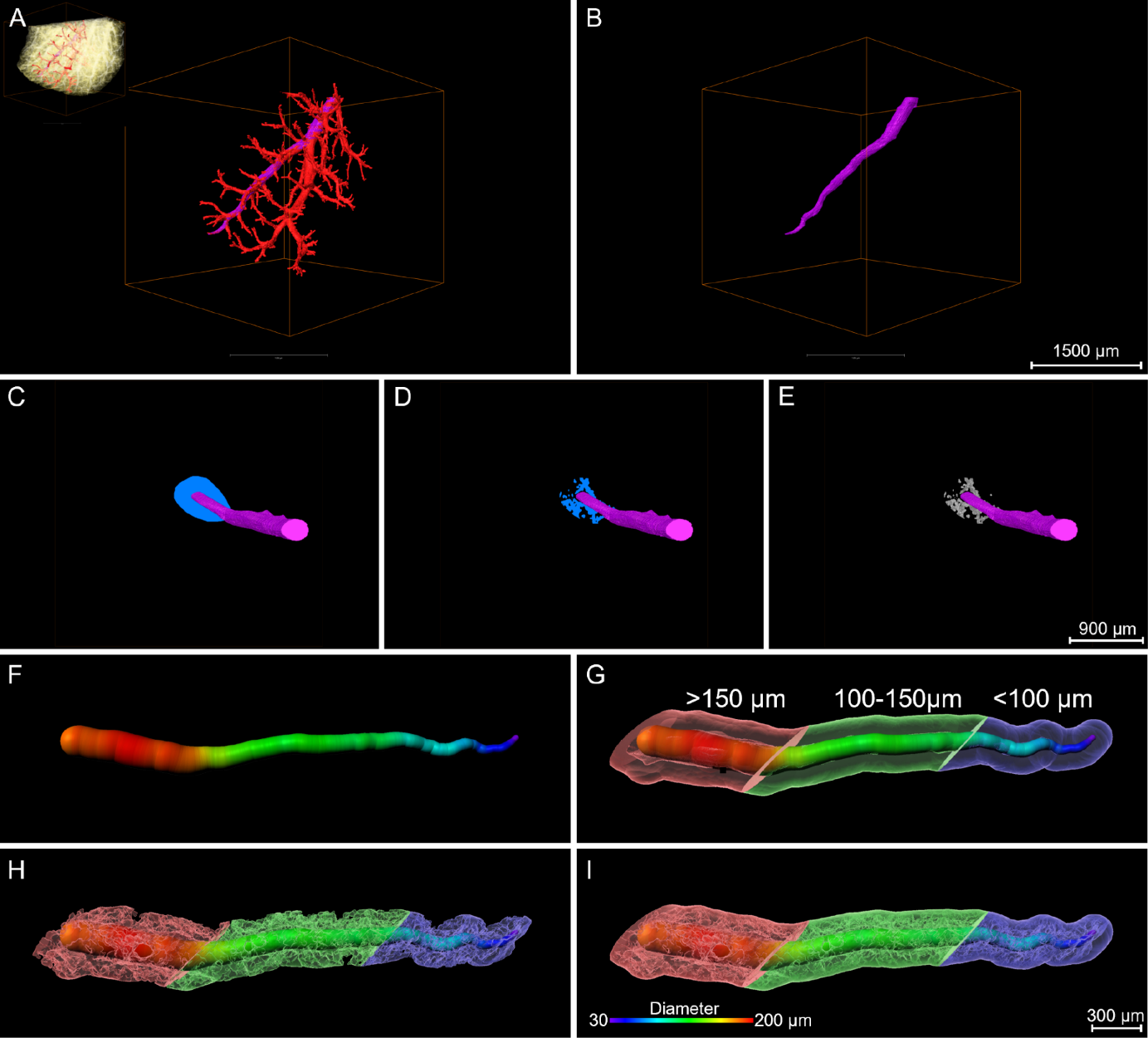


**Figure S9 Segmentation and quantitative analysis of the** **steatotic regions surrounding the central veins**

(**A, B**) Representative individual branch of central vein (purple color) segemented from the same data block as shown in Figure 6B,D. Scale bar, 1500 μm. (**C-E**) The regions surrounding the individual branch of central vein were obtained using dilation operation starting from the surface of central vein by 150 micrometers. The labels of the steatotic regions surrounding the central vein were achieved by multiplying the dilated labels in C and the labels segmented using deep learning model(D). The grayscale images of the steatotic regions surrounding the central vein were achieved by multiplying the labels in D and the raw grayscale images (E). Scale bar, 900 μm. (**F-I**) Quantitative analysis of the steatotic regions surrounding central veins of different diameter ranges:>150 μm, 100-150μm, <100 μm. The related data are presented in Table 3. Scale bar, 300 μm.

**Movie S1. 3D visualization of the steatotic regions of different distances from the central veins in different colors.**

At the very beginning, the video demonstrated the image of orthogonal slice (xy-axis) of the data block as shown in Figure 6B and Figure7C, L. And then, it showed two-dimensionally the different steatotic regions around the central veins with distinct colors: yellow (0 - 30 μm), red (30 -60 μm in red), blue (60 - 90 μm), and green (90 -120 μm). At last, the video exhibited the 3D reconstruction of these steatotic regions in different colors.
